# Antioxidant Activity, α-Glucosidase Inhibition and UHPLC–ESI–MS/MS Profile of Shmar (*Arbutus pavarii* Pamp)

**DOI:** 10.3390/plants10081659

**Published:** 2021-08-11

**Authors:** Nawal Buzgaia, Soo Yee Lee, Yaya Rukayadi, Faridah Abas, Khozirah Shaari

**Affiliations:** 1 Department of Chemistry, Faculty of Science, University of Benghazi, Benghazi, Libya; nawalbuz@gmail.com; 2 Natural Medicines and Products Research Laboratory (NaturMeds), Institute of Bioscience, University Putra Malaysia, Serdang 43400, Selangor, Malaysia; daphne.leesooyee@gmail.com (S.Y.L.); yaya_rukayadi@upm.edu.my (Y.R.); faridah_abas@upm.edu.my (F.A.); 3 Department of Food Science, Faculty of Food Science and Technology, University Putra Malaysia, Serdang 43400, Selangor, Malaysia; 4 Department of Chemistry, Faculty of Science, University Putra Malaysia, Serdang 43400, Selangor, Malaysia

**Keywords:** *Ericaceae*, *Arbutus pavarii* Pamp, antioxidant, α-glucosidase, plant phenolics, UHPLC–ESI–MS/MS

## Abstract

The genus *Arbutus* (*Ericaceae*) has been traditionally used in folk medicine due to its phytomedicinal properties, especially *Arbutus pavarii* Pamp. However, this plant has not been evaluated for its efficacy, quality, and consistency to support the traditional uses, potentially in treating diabetes. Despite previous studies that revealed the biological activities of *A. pavarii* as antioxidant and α-glucosidase inhibitory agents, scientific reports on the bioactive compounds that contribute to its health benefits are still scarce. Therefore, this research focused on the evaluation of antioxidant and α-glucosidase inhibitory activities of the methanol crude extracts and various fractions of the leaf and stem bark, as well as on metabolite profiling of the methanol crude extracts. The extracts and fractions were evaluated for total phenolic (TPC) and total flavonoid (TFC) contents, as well as the DPPH free radical scavenging, ferric reducing antioxidant power (FRAP), and α-glucosidase inhibitory activities. Methanol crude extracts of the leaf and stem bark were then subjected to UHPLC–ESI–MS/MS. To the best of our knowledge, the comparative evaluation of the antioxidant and α-glucosidase inhibitory activities of the leaf and stem bark of *A. pavarii*, as well as of the respective solvent fractions, is reported herein for the first time. Out of these extracts, the methanolic crude extracts and polar fractions (ethyl acetate and butanol fractions) showed significant bioactivities. The DPPH free radical and α-glucosidase inhibitions was highest in the leaf ethyl acetate fraction, with IC_50_ of 6.39 and 4.93 µg/mL, respectively, while the leaf methanol crude extract and butanol fraction exhibited the highest FRAP with 82.95 and 82.17 mmol Fe (II)/g extract. The UHPLC–ESI–MS/MS analysis resulted in the putative identification of a total of 76 compounds from the leaf and stem bark, comprising a large proportion of plant phenolics (flavonoids and phenolic acids), terpenoids, and fatty acid derivatives. Results from the present study showed that the different parts of *A. pavarii* had potent antioxidant and α-glucosidase inhibitory activities, which could potentially prevent oxidative damage or diabetes-related problems. These findings may strengthen the traditional claim on the medicinal value of *A. pavarii*.

## 1. Introduction

Medicinal plants have always been known as healthy and natural sources of combating drugs. Historically, for thousands of years, many of such plants have been used for treating various diseases [1]. The World Health Organization (WHO) reported that nearly 80% of the populations of developing countries rely on traditional medicine and consider medicinal plants as their primary sources of medication [2]. Plant extracts are mixtures, rich in natural product compounds, such as flavonoids, alkaloids, terpenoids, and tannins, many of which possess bioactive characteristics [3]. Common natural resources of these constituents are fruits, vegetables, herbs, spices, edible mushrooms, and a myriad of other examples, which make a huge contribution in our everyday diet. Similarly, there is a long list of medicinal plants that possess a wide array of therapeutic potentials, owing to the phytochemical diversity of the plant constituents. Plants with antioxidant and antidiabetic properties are among medicinal plants that have attracted a lot of research interests [4].

Antioxidants play an important role in maintaining the balance of free radicals resulting from metabolic processes or environmental sources and the antioxidant defense system of the body. An imbalance will lead to oxidative stress that can eventually cause several chronic diseases, such as cardiovascular disease, neurodegenerative disease, cancer, and type 2 diabetes [5]. There are several synthetic antioxidants, such as butylated hydroxytoluene (BHT), and butylated hydroxyanisole (BHA) that have been widely used as an antioxidant in food and pharmaceutical industries. However, the side effects of synthetic antioxidants, such as skin allergies, gastrointestinal tract problems, and increased risk of cancer [6,7], and consumer preferences to natural substances have diverted the attention of researchers to natural antioxidants [6,7,8]. 

One of the significant enzymes involved in carbohydrate digestion in the human body is α-glucosidase. This enzyme helps to release glucose from disaccharides or complex carbohydrates and raise postprandial blood glucose level [9]. One of the therapeutic approaches for the management of diabetes mellitus is the inhibition of this enzyme. By inhibiting α-glucosidase, carbohydrate digestion and glucose absorption can be slowed down, eventually leading to suppression of postprandial hyperglycemia. Several synthetic α-glucosidase inhibitors, such as metformin and acarbose, are available in the market to treat diabetes mellitus. However, these synthetic drugs have brought about unfavorable side effects to the diabetic patients, such as abdominal distention, flatulence, meteorism, and diarrhea [7]. Recent studies have shown the remarkable effectiveness of some phytochemicals in controlling diabetes mellitus [10]. Therefore, there is an increased interest of seeking natural-based drugs from plants that contain a substantial amount of α-glucosidase inhibitory compounds [11,12]. 

*Arbutus pavarii* Pam, an evergreen plant of the family *Ericaceae*, is vernacularly known as “Shmar” in the Libyan society [13,14]. This plant has been used in traditional medications for various ailments, including cold, tuberculosis, gastritis, kidney diseases, cancer, and renal contagions [15]. Studies have reported that *A. pavarii* contained compounds of various classes, namely hydroxyquinone (arbutin), triterpenoid (oleanolic acid, lupeol and α-amyrin) flavonoids (catechin, quercetin, dihydroquercetin, isoquercitrin, kaempferol, myricetin, rutin, naringin, neodiosmin, naringenin-7-*O*-glucoside, isovitexin-7-*O*-glucoside, and delphinidin-3-*O*-rutinoside), and phenolic acids (caffeic, ferulic, gallic, rosmarinic, chlorogenic, and salicylic acids) [15,16]. Moreover, *A. pavarii* has been reported to exhibit potential antioxidant and α-glucosidase inhibitory activities [17]. However, only the aerial parts of the plant as a whole was evaluated for these biological properties. The comparative evaluation of the different parts of the plant as well as their different fractions has yet to be explored. Furthermore, the data available regarding the bioactive compounds of different parts of this plant are also limited. Therefore, the main aim of this study was to evaluate the potential antioxidant and α-glucosidase inhibitory activities of the methanol crude extracts and various solvent fractions of *A. pavarii* leaf, and stem bark, as well as to characterize the phytochemical profile of the active samples. The findings of this study may enrich the knowledge regarding the therapeutic properties of *A. pavarii* and reveal its potential as natural source of antioxidants and α-glucosidase inhibitors. 

## 2. Results and Discussion

### 2.1. Total Phenolic Content (TPC)

The antioxidant potential of *A. pavarii* leaf and stem bark were first assessed by determination of their total phenolic and total flavonoid contents (TPC and TFC), followed by their antiradical activity towards 2,2-diphenyl-1-picrylhydrazyl (DPPH) and ferric reducing antioxidant power (FRAP). Although the use of colorimetric-based total phenolic and antioxidant assays for describing the bioactivity of chemical constituents in the absence of cell-based or in vivo test has been controversial, studies have revealed the positive correlation between results obtained from these colorimetric methods and cell-based assays [18,19,20]. Hence, these colorimetric methods are still essential screening tools for the assessment of antioxidant potential. The contents of phenolic compounds in the methanolic crude extracts and solvent fractions of *A. pavarii* leaf and stem bark were determined by using the Folin–Ciocalteu reagent [21] and the results are presented in Table 1. The leaf methanol crude extract contained 886.57 mg GAE/g extract of TPC while the TPC of its respective solvent fractions ranged from 62.54 to 790.76 mg GAE/g fraction. Among the four solvent fractions, the ethyl acetate fraction possessed the highest TPC (790.76 mg GAE/g fraction) followed by butanol (390.47 mg GAE/g fraction), chloroform (186.94 mg GAE/g fraction), and hexane fractions (62.54 mg GAE/g fraction). The results revealed that most of the phenolic compounds were distributed in the ethyl acetate fraction of the leaf methanol crude extract and suggested that the phenolic compounds are of moderate polarity. Meanwhile, the stem bark of *A. pavarii* also contained high TPC with 795.55 mg GAE/g extract. The TPC of its fraction ranged from 199.14 to 707.61 mg GAE/g fraction. However, unlike the leaf extract, the highest TPC of the stem bark methanol crude extract was in the butanol fraction (707.61 mg GAE/g fraction), followed by the ethyl acetate (480.21 mg GAE/g fraction), chloroform (322.68 mg GAE/g fraction) and hexane fractions (199.14 mg GAE/g fraction). This indicated that the phenolic constituents of the stem bark were mainly distributed in the butanol fraction and suggested that the compounds were of high polarity. The different trends in the results of the leaf and stem bark phenolic contents could be attributed to a different composition of the phenolic constituents of the different parts of the plant [22]. 

### 2.2. Total Flavonoid Content (TFC)

To determine the total flavonoid content (TFC) in the leaf and stem bark extract and solvent fractions of *A. pavarii*, a colorimetric approach based on flavonoid–aluminum chloride complexation was employed [23], and the results are presented in Table 1. The results revealed the presence of high flavonoid content in the leaf and stem bark extracts of *A. pavarii*. The methanol crude extract of *A. pavarii* leaf contained 442.06 mg QE/g extract while the TFC of its respective solvent fractions ranged from 58.21 to 369.52 mg QE/g fraction. The highest TFC was found in the ethyl acetate fraction, which contained 369.52 mg QE/g fraction, followed by butanol, chloroform and hexane fractions, which contained 277.72, 109.09, and 58.21 mg QE/g fraction, respectively. These results were of the same trend as the TPC results, suggesting that flavonoids could very well be the major class of phenolic constituents in the leaves of *A. pavarii*. 

On the other hand, the methanol crude extract of *A. pavarii* stem bark contained 638.93 mg QE/g of total flavonoid, which was significantly higher than in the leaf. The difference in the TFC of the leaf and stem bark could be due to the production and accumulation of different secondary metabolites in the leaf and stem bark [24]. Besides, similar to the fractions of the leaf extract, the ethyl acetate fraction of the stem bark extract contained the highest TFC with 707.61 mg QE/g fraction, followed by butanol, chloroform, and hexane fractions, with 213.32, 204.83, and 38.41 mg QE/g fraction, respectively. These results were different than that of the TPC results. Although it contained the highest TPC, the butanol fraction had relatively lower TFC, suggesting that it contains phenolic compounds other than flavonoids, which also revealed the diversity of phenolic compounds present in *A. pavarii* stem bark [25]. 

### 2.3. 2,2-diphenyl-1-picrylhydrazyl (DPPH) Radical Scavenging Activity 

The free radical scavenging activity of the plant extracts and their respective fractions was determined using the DPPH free radical scavenging assay [21]. The presence of antioxidants leads to the reduction of the DPPH free radicals, and hence the dark violet-colored solution is transformed to yellow. Table 1 show the DPPH free radical scavenging activity of the leaf and stem bark of *A. pavarii*, expressed as IC_50_ values. The leaf methanol crude extract inhibited the DPPH free radicals with IC_50_ value of 17.57 µg/mL. For the leaf solvent fractions, the ethyl acetate fraction exhibited the most potent DPPH scavenging activity, with IC_50_ value of 6.39 µg/mL. This was followed by butanol, chloroform, and hexane fractions, with IC_50_ values of 27.69, 39.50, and 95.82 µg/mL, respectively. It is noteworthy that the IC_50_ value of the ethyl acetate fraction was even lower than that of quercetin (IC_50_ = 8.60 µg/mL), which was used as positive control in the assay. The more potent DPPH scavenging activity of the ethyl acetate fraction as compared to the methanolic crude extract could be explained by the high concentration of free radical scavenging compounds in the fraction after the fractionation process. In addition, the potent DPPH scavenging activity of the ethyl acetate fraction could be contributed by the presence of high amount of phenolic compounds. A positive relationship between phenolic content and DPPH scavenging activity has been reported in previous studies [19,20,26]. 

Meanwhile, the stem bark methanol crude extract exhibited high DPPH scavenging activity with an IC_50_ value as low as 8.67 µg/mL. Among its solvent fractions, the ethyl acetate and butanol fractions showed the more potent activities, with IC_50_ values (8.35 and 8.71 µg/mL, respectively) close to that of the methanol crude extract, and comparable with that of quercetin (IC_50_ = 8.60 µg/mL). The potent activity exhibited by both the ethyl acetate and butanol fractions indicated that the constituents distributed in ethyl acetate and butanol fractions are structurally effective for scavenging DPPH free radicals. While the phenolic compounds could be the DPPH free radical scavengers in these fractions, the activity of the stem bark butanol fraction could be contributed by phenolic compounds with relatively higher polarity, perhaps those with more polar functional groups or sugar attachment. In addition, the DPPH scavenging activity of stem bark is also more significant as compared to the leaf of *A. pavarii*, as revealed by the lower IC_50_ value of the stem bark. The better activity of the stem bark could be possibly due to its more diverse phenolic compositions. 

### 2.4. Ferric Reducing Antioxidant Power (FRAP)

The ferric reducing antioxidant power (FRAP) assay allows the examination of the reducing power of samples. The reducing ability of a sample may reflect its electron transferring capability, which is an important mechanism of antioxidants [27,28,29,30] The FRAP values (mM Fe (II) equivalent) of *A. pavarii* leaf and stem bark extracts, as well as their respective fractions have been calculated by constructing a standard curve between the absorbance and the concentration of FeSO_4_ standard [31]. The results are shown in Table 1. The *A. pavarii* leaf methanolic extract exhibited reducing power with FRAP value of 82.95 mM Fe (II)/g extract, while the FRAP values of its fractions ranged from 46.76 to 86.33 mM Fe (II)/g extract. Unlike the results of the aforementioned assays, both the ethyl acetate and butanol fractions of *A. pavarii* leaf exhibited almost similar reducing power and are close to the FRAP value of the methanolic extract. The results of the stem bark showed the same trend as the leaf, although overall it showed slightly lower reducing power than the leaf. These results indicated the presence of strong electron donating antioxidants in the extracts as well as the ethyl acetate and butanol fractions which reduced ferric ions into ferrous ions under the reaction conditions [29]. The phenolic compounds, both flavonoids and non-flavonoid compounds, could be responsible for the reducing ability of *A. pavarii* leaf and stem bark. This is in agreement with previous studies that reported significant correlations between both TPC and TFC, and FRAP of grape by-products [19], and *Clinacanthus nutans* [26]. 

### 2.5. α-Glucosidase Inhibitory Activity 

According to the results of α-glucosidase inhibitory activity of *A. pavarii* leaf and stem bark extracts and fractions, which are presented in Table 1, the leaf methanolic extract inhibited the α-glucosidase enzyme with IC_50_ value of 8.75 µg/mL. However, for the leaf fractions, the ethyl acetate fraction exhibited the most potent inhibitory activity with IC_50_ value of 4.93 µg/mL, followed by butanol (IC_50_ value 10.44 µg/mL) and chloroform (IC_50_ value 62.64 µg/mL) fractions. The IC_50_ of hexane fraction was not able to be determined as the inhibition against the α-glucosidase enzyme was less than 50% at all the concentrations used. On the other hand, the stem bark methanolic extract inhibited the α-glucosidase enzyme with IC_50_ value of 6.78 µg/mL, which was significantly more potent as compared to the leaf methanol extract. For stem bark fractions, the trend was found to be similar to the fractions of the leaf methanolic extract, with the exception at the insignificant different of the activity of ethyl acetate and butanol fractions. Moreover, based on the IC_50_ values, the ethyl acetate fractions of both leaf and stem bark, and the butanol fraction of stem bark showed higher α-glucosidase inhibitory activity compared to quercetin, which was used as positive control in the assay. These results revealed an increasing activity with an increasing polarity of the fraction, which could be explained by the existence of highly polar compounds. In other words, it can be due to the amount of phenolic compounds and the type of phenolics present in the sample that may be responsible for the strong inhibition activity against α-glucosidase enzyme. The results obtained in the present study are in good agreement with previous work, which reported the potency of phenolic rich samples in inhibiting α-glucosidase enzyme, in addition to strong antioxidant activity [26]. Besides, previous research has also reported the increased inhibitory effect against α-glucosidase enzyme with increasing polarity of the plant extracts or fractions, with more polar and lower molecular weight phenolic constituents, such as phenolic acids as inhibitors of the enzyme [32]. Furthermore, various studies have outlined that the *Arbutus* genus could be a great natural source of phenolic and flavonoid compounds which are well known to have a strong hypoglycemic potential [15]. To the best of our knowledge, this is the first report on α-glucosidase inhibitory effect of leaf, stem bark extracts, and fractions of *A. pavarii*. Its potential therapeutic use for treating or managing diabetes could be worthy of further pharmacological investigations.

### 2.6. Putative LCMS Profiles of A. pavarii Crude Leaf and Stem Bark Methanol Extracts

**Figure 1:**
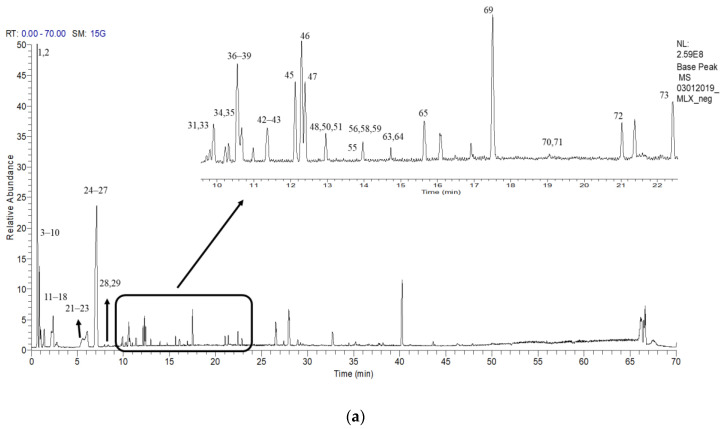

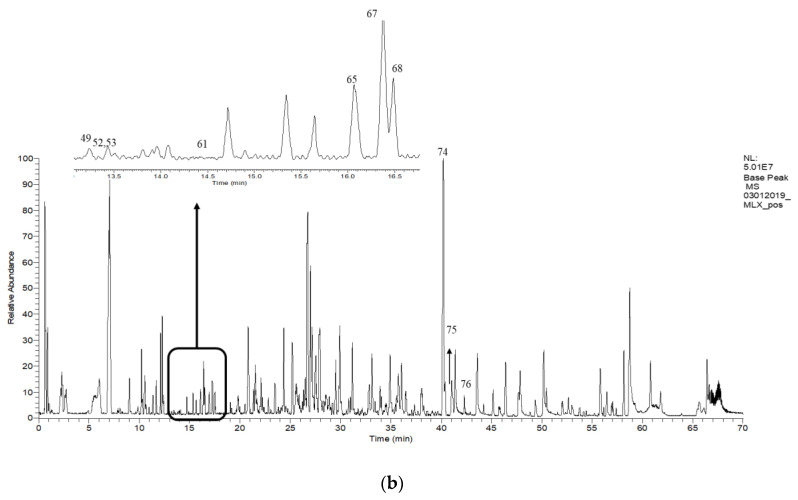
LCMS base peak chromatogram of *A. pavarii* leaf methanol extract in negative (**a**) and positive (**b**) ionization modes.

**Figure 2:**
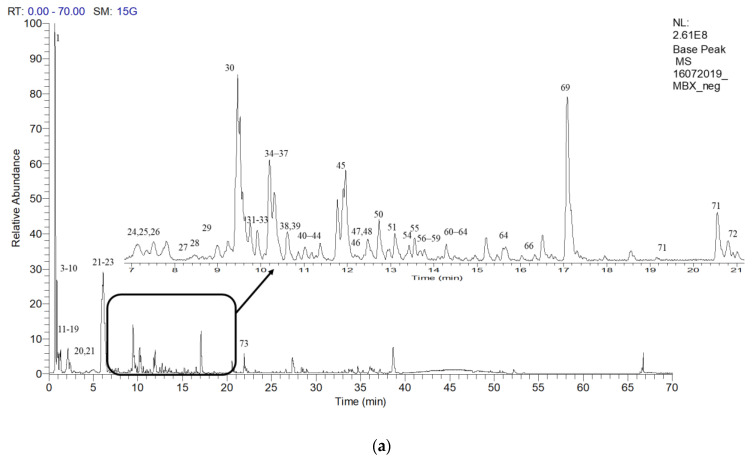

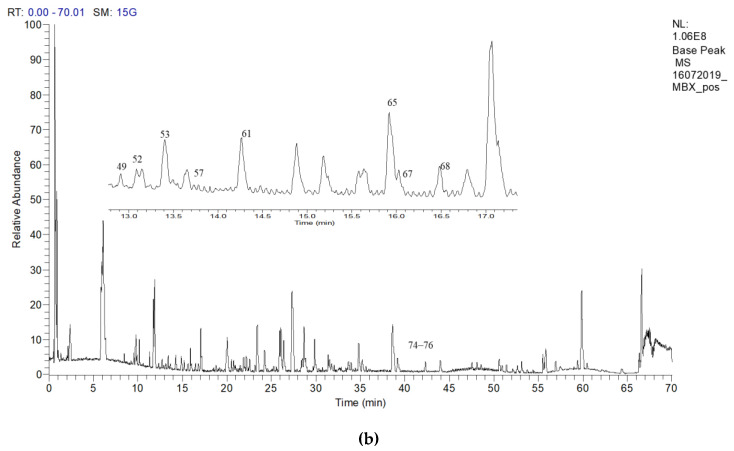
LCMS base peak chromatogram profiles of methanol of *A. pavarii* stem bark extract in negative mode (**a**) and positive mode (**b**).

Analyses of medicinal plants have benefited from the application of liquid chromatography coupled with mass spectrometry (LC–MS) due to the increasingly improved separation and detection abilities of the instruments [33]. The leaf and stem bark methanol extracts of *A. pavarii* showed good activities in all assays (antioxidant and α-glucosidase inhibitory activities). Hence, these extracts were further characterized using LC–MS/MS to gain better insight into the components that may be contributing to the studied activities. The base peak chromatograms of *A. pavarii* leaf and stem bark methanol extracts are displayed in Figure 1; Figure 2, respectively; while Table 2 summarizes the retention time (Rt), ionization mode (−ve/+ve), experimental and theoretical parent ion (*m*/*z*), error (ppm), MS/MS data, and presence of the identified compounds. A total of 76 compounds were putatively identified based on the MS/MS data in comparison with literature. The base peak chromatograms showed that most of the prominent peaks were attributed to the presence of phenolic compounds. This could support the high TPC and TFC values, and hence the potent antioxidant and α-glucosidase inhibitory activities of the *A. pavarii* leaf and stem bark.

#### 2.6.1. Identification of Phenolic Acid and Phenolic Acid Glycoside Derivatives

In this work, compounds that are putatively identified as phenolic acids and phenolic acid glycoside derivatives were classified as either gallic acid and its derivatives, or other phenolic acids and glycoside derivatives. Compounds **3**, **4**, **5**, and **8** were identified as isomers of gallic acid monoglucoside. They had pseudomolecular ion at *m*/*z* 331.0671, *m*/*z* 331.0675, *m*/*z* 331.0672, and *m*/*z* 331.0667, respectively. Their fragment ion at *m*/*z* 169.01 [M-H-162] is due to the neutral loss of a glucosyl moiety (162 Da) (Table 2). This agrees with previous reports [34,35]. Compounds **6**, **7,** and **15** showed a pseudomolecular ions at *m*/*z* 169.0134, 343.0668, and 325.0566, respectively, in negative mode and were putatively assigned as gallic acid and gallic acid derivatives. Their fragment ions at *m*/*z* 169.01 by the neutral loss of a shikimic acid [M-H-156] and a quinic acid [M-H-191], and fragment ion at *m*/*z* 125.02 by the neutral loss of CO_2_ (44Da), which are characteristic MS fragments of a gallic acid moiety [34,35,36] made these compounds identified as gallic acid, galloyl shikimic acid, and galloyl quinic acid, respectively. 

Compound **10** with a pseudomolecular ion of *m*/*z* 315.0721, at Rt = 1.96 min was identified as protocatechuic-*O*-glucoside with molecular formula C_13_H_16_O_9_. It produced fragment ion at *m*/*z* 153.02 which corresponding to protocatechuic acid after the neutral loss of the glucosyl moiety [M-H-162]. The fragment ion at *m*/*z* 109.03 [M-H-162-44] indicates a further loss of CO_2_ [37]. Compounds **11** and **12** in the leaf, with pseudomolecular ions at *m*/*z* 329.0878 and *m*/*z* 329.0875 at retention time 1.98 and 2.04 min, respectively, were identified as vanillic acid-*O*-glucoside ester isomers. The fragment ion at *m*/*z* 167.03 was due to the loss a glucosyl moiety and the fragmentation was in agreement with the Morales-Soto et al. [38]. Compound **18** showed a pseudomolecular ion of *m*/*z* 359.0981, and it was identified as syringic acid-*O*-glucoside and detected in both leaf and stem bark extracts. The MS/MS data showed a fragment ion of *m*/*z* 197.04 [M-H-162] due to the loss of glucosyl moiety, also the ion at *m*/*z* 197.04 was the syringic acid moiety. It further loses a carbon dioxide ion to give *m*/*z* 153.05 [M-H-162-44] [37].

Compounds **26** and **36** showed a pseudomolecular ion at *m*/*z* 483.0776 and 635.0889, respectively and were detected in the leaf and stem bark. They exhibited a fragmentation pattern similar to that of monogalloyl glucose. However, their base peak at *m*/*z* 169.01 indicated the neutral loss of two (324 Da) [M-H-162-162] and three glucosyl residues (486 Da) [M-H-162-162-162], respectively. Hence, they were identified as digalloyl and trigalloyl glucose. Their fragmentation patterns were in agreement with the reports of Abu-Reidah et al. and Liu and Seeram et al. [35,36]. Compound **47** showed a pseudomolecular ion at *m*/*z* 300.9984 in negative mode at Rt = 1.96 min (C_14_H_6_O_8_). It was assigned as ellagic acid based on comparison with the previous report [36]. 

#### 2.6.2. Identification of Flavonoids and Derivatives

Flavonoids (C6–C3–C6) are phytoconstituents, which contain 15 carbons with two aromatic rings associated by a three-carbon bridge. Based on hydroxylation and different functionalities in chromane (ring C), these polyphenols are further divided into flavones, flavonols, flavan-3-ols, isoflavones, flavanones, and anthocyanidins [39]. In plants, these substances work as regulatory compounds, colorants, and protecting the newly developed plants cells against UV light, wound, pathogens, and herbivores [40,41]. Furthermore, flavonoids are famous for antibacterial, antioxidants, antidiabetics, and various other bioactive activities that have been interested due to its benefits for human health, curing, and preventing of many serious diseases [8,42]. The current study is the first report for the identification of flavonoids in the stem bark of *A. pavarii*.

##### Identification of Flavan-3-ols and Derivatives

Analysis of the LCMS profiles of *A. pavarii* leaf and stem bark methanol extracts indicate the presence of monomers, dimers, trimers, and tetramers of proanthocyanidin (PAs). Three compounds (**13**, **24**, and **32**) were putatively identified as PA monomers. Compound **13** was identified as (+)-gallocatechin or (-)-epigallocatechin. It displayed a pseudomolecular ion at *m*/*z* 305.06638 in the negative mode. This compound generated fragment ions at *m*/*z* 167.03 and 137.02 by retro-diels-alder fragmentation (RDA). Fragment ion at *m*/*z* 138.03 was formed by benzofuran fission (BFF) fragmentation of ion [M-H-C_7_H_6_O_3_]-, while ion at *m*/*z* 125.02 was obtained by heterocyclic ring fragmentation (HRF). Fragment ion at *m*/*z* 179.03 was due to the loss of trihydroxybenzene moiety and fragment ion at *m*/*z* 261.08 corresponded to a loss of C_2_H_4_O [43]. Compounds **24** and **32** showed pseudomolecular ions at *m*/*z* 289.0714 at time 7.01 min and 289.0717 at 9.86 min in the negative mode. Based on the elution order in previous report, compounds **24** and **32** were identified as catechin and epicatechin, receptively [44]. They exhibited the similar MS/MS fragmentation. Fragment ions at *m*/*z* 165.02 were resulted from HRF fragmentation, while *m*/*z* 151.04 and 137.02 from RDA fragmentation. Besides, the fragments at *m*/*z* 271.06 and 245.08 resulted from the loss of water and carbon dioxide, respectively [45,46]. 

Compounds **16**, **20**, **23,** and **50** were putatively identified as the derivatives of PA monomers. Compounds **16**, **20,** and **23** exhibited pseudomolecular ions at *m*/*z* 451.1250, 451.1254 and 451.1241 in the negative mode with different retention times at 2.60, 5.40, and 6.81 min, respectively. They exhibited the loss of 162 Da to give fragment ions at *m*/*z* 289.07, which corresponds to catechin or epicatechin. The fragment ions at *m*/*z* 245.08, 151.04 and 125.02 revealed the subsequent fragmentation of the catechin or epicatechin aglycone. These compounds were identified as the (epi)catechin-3-*O*-glucoside isomers after the comparison of their MS/MS fragmentation patterns with those reported previously [47,48,49]. Compound **50** was identified as (epi)catechin-3-*O*-gallate. It displayed a pseudomolecular ion at *m*/*z* 441.0825 in the negative mode at retention time 12.97 min. Its fragmentation showed a single ion [M-H-152]^-^ at *m*/*z* 289.07, which corresponded to a loss of deprotonated galloyl moiety [50].

Ten compounds (**9**, **17**, **21**, **22**, **31**, **38**, **42**, **43**, **55,** and **56**) were putatively identified B-type PA dimers in the leaf and the stem bark of *A. pavarii*. The two flavan-3-ol units was defined as the top unit and the base unit to describe the structure of the PA dimer. The most common linkage is the B-type linkage between C4 of the top unit and C8 of the lower unit (noted C4→C8) or between C4 of the top unit and C6 of the lower unit (C4→C6). The heterocyclic ring of the flavan-3-ol fragments through the processes of RDA, HRF, and quinone methide (QM) cleavage. These processes can assist to determine the structure and assigning the link sequence of the monomeric units [51].

**Scheme 1:**
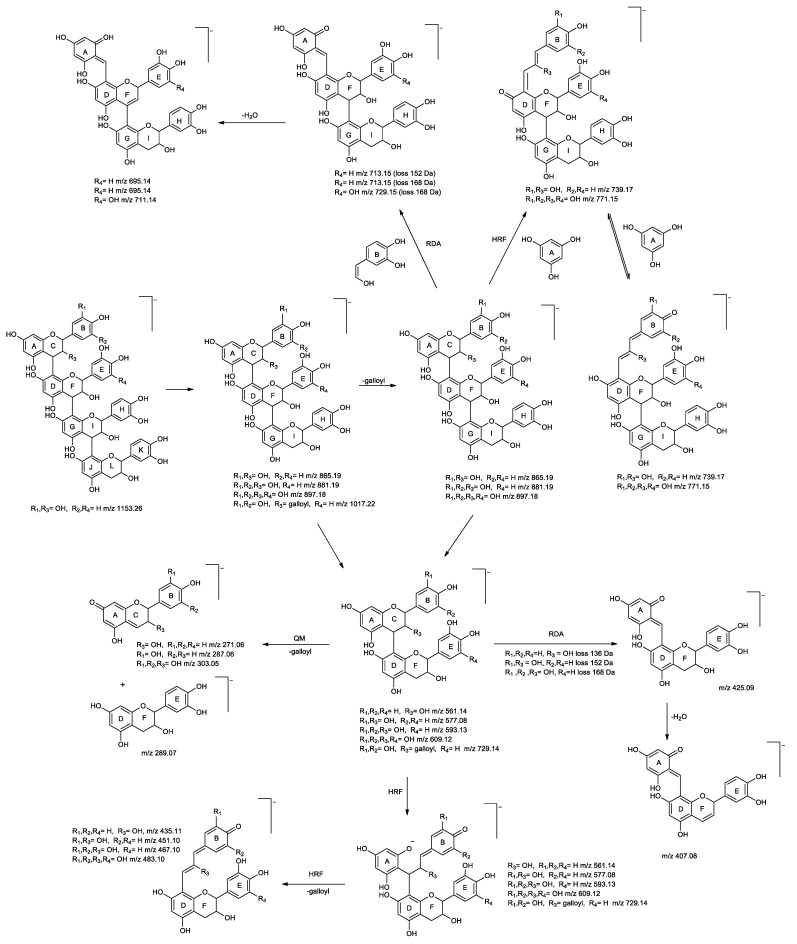
Fragmentation pathways of proanthocyanidin dimers, trimers, and tetramers shown in negative ion mode.

Compound **9** showed a pseudomolecular ion at *m*/*z* 609.1244 in negative ionization mode, suggesting a molecular formula of C_30_H_26_O_14_. This compound was identified as (epi)gallocatechin + (epi)gallocatechin. The characteristic fragmentation patterns common for PA dimers could be observed in its MS/MS spectra. The fragment ion at *m*/*z* 441.08 was generated by the RDA cleavage (loss of 168 Da), while a successive loss of water molecule (18 Da) gave rise to the ion *m*/*z* 423.07. The fragment ion at *m*/*z* 305.07, which corresponded to deprotonated (epi)gallocatechin [(epi)gallocatechin-H]^−^ was a result of the QM cleavage of the interflavan bond. The presence of fragment ions at *m*/*z* 483.10 and *m*/*z* 125.02 may be ascribed to the HRF on the top unit of the dimer. Compounds **22**, **31,** and **42**, having the same fragmentation patterns as compound **9**, were identified as (epi)catechin + (epi)catechin isomers. The fragmentation pathways are illustrated in Scheme 1 [45,52]. 

Compounds **17** and **21** were identified as (epi)gallocatechin + (epi)catechin isomers, with the pseudomolecular ions at *m*/*z* 593.1291 and *m*/*z* 593.1314. Both isomers exhibited similar fragmentation pattern. The fragment at *m*/*z* 441.08 was an indication of the galloyl moiety loss [M-H-152]^−^ while the fragment ion at *m*/*z* 407.08 [M-H-168-18] was produced via RDA mechanism and successive loss of water molecule. These fragments are characteristic for (epi)gallocatechin. Furthermore, the RDA reaction on the top unit of dimer has been reported to be more energetically favorable, due to the formation of larger π-π hyperconjugated system [53]. Hence, (epi)gallocatechin was suggested as the top unit of this dimer. The (epi)catechin as the base unit was further confirmed by the presence of the fragment ion at *m*/*z* 289.07, which was the result of QM cleavage. The ion *m*/*z* 287 would presence if the (epi)catechin was the top unit [53]. Besides, the fragments at *m*/*z* 125.02 and *m*/*z* 467.10 were attributed to the HRF on the top unit. The HRF on the top unit was also more favorable due to the same reason as the RDA [54]. With the same principle applied, compounds **38**, **43** and **55** were identified as isomers of (epi)catechin gallate + (epi)cat, while compound **56** was assigned as (epi)afzelechin + (epi)catechin. The fragmentation pathways of these compounds are also illustrated in Scheme 1 [55]. 

Eight compounds (**14**, **19**, **25**, **28**, **30**, **33**, **39**, and **44**) were putatively identified B-type PAs trimeric in the leaf and the stem bark of *A. pavarii*. Compound **14** has a pseudomolecular ion of *m*/*z* 897.1866 in negative ionization mode. This compound was identified as the (epi)gallocatechin + (epi)gallocatechin + (epi)catechin. The ion peak at *m*/*z* 729.15 loss of (168 Da) corresponds to the RDA cleavage from the top unit of the trimer and was followed by water molecule loss (*m*/*z* 711.14). Other fragment ions were detected, resulting from dimeric fragment at *m*/*z* 593.13 corresponding to the [(epi)gallocatechin + (epi)catechin-H]^−^ fragment and the [(epi)catechin-H]^−^ fragment with *m*/*z* 289.07 were obtained (Scheme 1). This PA must have (epi)gallocatechin as the top and the middle units and (epi)catechin as the base unit [56]. Having the same fragmentation patterns as compound **14**, compounds **19** and **30** were identified as (epi)gallocatechin + (epi)catechin + (epi)catechin isomers [57], while compounds 25, 28, and 39 were identified as (epi)catechin + (epi)catechin + (epi)catechin isomers [58,59,60]. Fragmentation pathways of these compounds are illustrated in Scheme 1 as well. 

Compounds **33**, **34,** and **44** (Rt = 9.99, 10.30, and 11.62 min, respectively) revealed a pseudomolecular ions of *m*/*z* 1017.2080, *m*/*z* 1017.2085 and *m*/*z* 1017.2098 in negative mode. These compounds were identified as (epi)catechin gallate + (epi)catechin + (epi)catechin isomers. Compared with literature, fragmentation of these compounds produces characteristic ions at *m*/*z* 865.19, 577.13, 407.08, and 289.07. The fragment ion at *m*/*z* 865.19 [M-H-152]^−^ was due to the galloyl moiety loss as illustrated in Scheme 1 [61,62,63].

Three isomers were putatively assigned as B-type tetrameric PA in the methanol extract of the stem bark. Compounds **27**, **37,** and **41** showed pseudomolecular ions at *m*/*z* 1153.2627, *m*/*z* 1153.2617, and *m*/*z* 1153.2635, respectively, in the negative mode with molecular formal C_60_H_50_O_24_. These compounds produced similar fragmentation patterns with similar intensities of ions. Based on the presence of fragment ions at *m*/*z* 865.20, 577.14, 407.08, and 289.07, they were identified as ((epi)catechin + (epi)catechin + (epi)catechin + (epi)catechin isomers. Fragmentation pathway is shown in Scheme 1 [62,63].

##### Identification of Anthocyanins and Derivatives

Anthocyanins are categorized as a class of flavonoids, and they are known for their beneficial effects on both humans and animals. They are usually pigments that give colors to many plants, including *A. pavarii*. They are present in nature as glycosides [64]. One of the most notable and distinguishing features of anthocyanins group is the ability of its structure to change under distinct pH conditions, leading to a change of color [65]. In this study, the anthocyanins were identified in the methanol extracts of leaf and stem bark of *A. pavarii* in the positive ionization mode. The fragmentation of the [M+H] ion allows the identification of the anthocyanin aglycone and the glycone. Seven anthocyanins derivatives were putatively assigned in leaf and stem bark of the *A. pavarii* (compounds **49**, **52**, **53**, **61**, **65**, **67,** and **68**). They showed the characteristic fragment ions of delphinidin, cyanidin, petunidin, peonidin, and malvidin aglycones at *m*/*z* 303.05, 287.05, 317.06, 301.07, and 331.07, respectively. Five of these anthocyanins had glucose attached to the aglycon [M+H-162]^+^ and one had coumaroyl glucose [M+H-308]^+^ attachment. The assignments of these compounds were in good agreement with those previously reported [64,66,67,68].

##### Identification of Flavonol

In both leaf and stem bark methanol extracts, a total of seven compounds (**45**, **48**, **51**, **58**, **62**, **64,** and **69**) were putatively identified as quercetin derivatives based on the presence of aglycone fragment ion at *m*/*z* 301 corresponding to the [Y_0_]^−^ and the characteristic fragment ions at *m*/*z* 271 and 151 in their MS/MS spectra. The characteristic fragment ions at *m*/*z* 271.02 and 255.03 were due to the loss of [Y_0_-CHO]^−^ and [Y_0_-H_2_O-CO]^−^, respectively. The fragment ions at *m*/*z* 179.00 and 151.00 showed the characteristic RDA cleavage of C-ring. All these fragment ions led to the identification of aglycone as quercetin (compound **69**, *m*/*z* 301.0353). In addition, for the flavonol mono-*O*-glycosides in which the glycosylation takes place at the 3-position, the [Y_0_-H]^−^ ion will be significantly higher than that of the [Y_0_]^−^ ion [69]. This can be observed in the compounds **51**, **58,** and **62** where the [Y_0_-H]^−^ ion at *m*/*z* 300.03 was more profound than the [Y_0_]^−^ ion at *m*/*z* 301. Transitions of pseudomolecular ions of these compounds to the [Y_0_-H]^−^ ion revealed the loss of glucosyl (*m*/*z* 162), arabinosyl/xylosyl (*m*/*z* 146) and rhamnosyl (*m*/*z* 132) moieties, respectively. Hence, compounds **51**, **58,** and **62** were identified as quercetin-3-*O*-glucoside, quercetin-3-*O*-(arabinoside/xyloside) and quercetin-3-*O*-rhamnoside, respectively [69,70].

Compound **45** with a pseudomolecular ion at *m*/*z* 615.0997 in negative mode was assigned as quercetin-*O*-galloyl-hexoside. Its MS2 fragment ion at *m*/*z* 463.09 was due to the neutral loss of gallic acid (169 Da) while the neutral loss of 331 Da (loss of galloyl and hexose moieties) produced the [Y_0_]^−^ ion at *m*/*z* 301.03. The fragment ion [Y_0_-H]^−^ at *m*/*z* 300.03, coupled with the RDA fragments of *m*/*z* 151.00 and *m*/*z* 179.00 indicates that it was a quercetin galloyl-glucoside. This observation was similar to those reported by Mendes et al. [34].

Compound **48**, with the pseudomolecular ion at *m*/*z* 609.1456 in negative mode was identified as quercetin-*O*-diglycoside. A neutral loss of 308 Da (loss of a pentose *m*/*z* 146 and a hexose *m*/*z* 162 moieties) was observed in its MS2 fragmentation. Besides, the presence of the abundant [Y_0_-H]^−^ ions in the mass spectrum indicates the loss of sugar moieties from the 3-*O* position. Therefore, compound 48 was characterized as flavonol 3-*O*-diglycosides. It was identified as rutin (quercetin-3-*O*-rutinoside) because it exhibited the mass spectrometric behavior of diglycoside which showed a C1→C2 connection between the two monosaccharides [69]. 

Compound **64** showed a pseudomolecular ion at *m*/*z* 583.1099 in negative mode with molecular formula C_28_H_24_O_14_ was assigned as the quercetin-*O*-(p-hydroxy) benzoyl-hexoside. This compound showed a base peak at *m*/*z* 300.03 [Y_0_-H]^−^. The fragment ion at *m*/*z* 463.09 indicates a loss of 120 Da which presumably corresponds to the loss of hydroxybenzoyl ion [M-H-hydroxybenzoyl]^−^, while a further glucosyl moiety loss lead to the formation of [Y_0_]^−^ ion at *m*/*z* 301.03 ([M-H-hydroxybenzoyl-Glc]^−^) [57].

In this current study, three derivatives of kaempferol were putatively identified in the methanol extracts of the leaf and stem bark. Compound **35** was identified as dihydrokaempferol-3-*O*-glucoside. It showed a pseudomolecular ion at *m*/*z* 449.1087 in negative mode at 10.35 min with molecular formula C_21_H_22_O_11_. This compound showed a fragment ion at *m*/*z* 287.06 [M-H-162]^−^ indicating the loss of hexose moiety. Further loss of water molecule produced the fragment *m*/*z* 269.05 [M-H-162-18]^−^ while RDA produced fragment ions at *m*/*z* 151.00 and 107.01. This agrees with previous reports of Abu-Reidah et al. [35]. Compounds **54** and **59,** with pseudomolecular ions at *m*/*z* 593.1520 and 447.0936, were identified as kaempferol 7-*O*-rhamnosyl-(1→6)-glucoside and kaempferol-3-*O*-glucoside, respectively. Both compounds generated fragment ions at *m*/*z* 285.04 due to the loss of sugar, 255.03 [Y_0_-CHO]^−^ and 227.03[Y_0_-H_2_O-CO]^−^. The fragments of *m*/*z* 179.00 and 151.00 were arising from RDA cleavage of C-ring [34,69,71].

Four compounds (**29**, **40**, **46,** and **60**) were detected and putatively assigned as myricetin derivatives in the leaf and stem bark of *A. pavarii.* Compound **29** was identified as dihydromyricetin. It showed a pseudomolecular ion at *m*/*z* 319.0462 (Rt = 9.00 min) in negative ionization mode. Furthermore, it showed fragment ion at *m*/*z* 301.04 due to the water loss [M-H-18]^−^, *m*/*z* 151.00 due to RDA C-ring cleavage and *m*/*z* 193.01 and *m*/*z* 125.02 due to the bond cleavage between C2−C1′. Moreover, the ions at *m*/*z* 165.02 and 153.02 were produced from the bond cleavage between C2−C3 and C9−O1, respectively. Successive losses of CO from the ion *m*/*z* 165.02 led to the formation of ions *m*/*z* 137.02 and 109.03. These fragmentations were in good agreement with those reported by Abu-Reidah et al. and Fan et al. [35,72]. Compounds **40**, **46**, and **60** were identified as myricetin and its derivatives based on the presence of fragment ion at *m*/*z* 316.02, 179.00, and 151.00 in the MS/MS spectra, which were corresponding to the myricetin aglycone and its subsequent fragment ions [69,73,74]. Compounds **40** and **46**, with pseudomolecular ions at *m*/*z* 479.0828 and *m*/*z* 463.0878, were assigned as myricetin3-*O*-glucoside and myricetin-3-*O*-rhamnoside, respectively. The deprotonated aglycone peaks observed at *m*/*z* 316.02 [M-H-162]^−^ were due to loss a glucosyl and rhamnosyl moieties, respectively. This type of cleavage suggests that the glycosylation took place at the 3-position based on the characteristic fragment ion of [Y_0_-H]^−^ [34,69]. The mass spectrum also provides more information about the aglycone which were the characteristic ions at *m*/*z* 287.02 [Y_0_-CHO]^−^ and 271.02 [Y_0_-H_2_O-CO]^−^. Compound **60** in the stem bark, with a pseudomolecular ion of *m*/*z* 317.0310 in negative mode was identified as myricetin. Fragment ions at *m*/*z* 179.00 and *m*/*z* 151.00 were due to RDA cleavage of C-ring [69,73,74].

Compound **57**, with a pseudomolecular ion at *m*/*z* 477.1042 was identified as isorhamnetin-3-*O*-glucoside and was detected in the stem bark. It lost a glucosyl moiety, producing the fragment at *m*/*z* 315.04 [M-H-162]-, and further loss of a CO_2_ produced the fragment at *m*/*z* 271.02. The fragment ions at *m*/*z* 285.04 due to loss of methoxy moiety [M-H-162-OCH_3_]^−^. The ion at *m*/*z* 271.02 produced the ion at *m*/*z* 243.03 via loss of the CO. Moreover, fragment ion at *m*/*z* 151.00 was due to RDA cleavage of C-ring. These fragmentations agree with those reported by Downey and Rochfort. [64]. Compound **66** has a pseudomolecular ion of *m*/*z* 435.1295 at Rt = 16.21 min. It was identified as phloridzin due to the fragmentation pattern, which agrees with the reports by Sánchez-Rabaneda et al. and Kumar et al. [75,76]. The fragment ion at *m*/*z* 273.08, which was due to the loss of a hexosyl moiety [M-H-162]^−^ and fragment ion of *m*/*z* 167.0351 resulted from the cleavage between C-α and C-β, which is characteristic ion for A-ring substitution [75,76,77].

#### 2.6.3. Identification of Triterpenoid Derivatives

Both compounds **74** and **75** in leaf and stem bark, which were detected at different retention times at 40.99 min and 41.17 min were putatively identified as triterpenoid isomers, they have pseudomolecular ions at *m*/*z* 457.3679 and *m*/*z* 457.3653, respectively, in positive ionization mode. Both compounds had the fragment ions *m*/*z* 439.35 [M+H-H_2_O]^+^, 411.36 [M+H-COOH]^+^ and 393.35 [M+H-H_2_O-COOH]^+^. In addition, compound **75** also showed fragment ions at *m*/*z* 249.18 (C_16_H_24_O_2_), 203.17 (-C_15_H_23_), and 133.10 (-C_10_H_13_), which were due to RDA fragmentation. A typical RDA fragmentation can be used to identify the presence of 12–13 double bonds in triterpenes (Δ^12^—ursine) [78]. Identification of these two compounds was carried out by comparing their mass spectra and elution order with the literature [79,80,81]. The first eluted compound **74** was identified a betulinic acid while compound **75,** which was eluted later was identified as ursolic acid. Compound **76** (Rt = 42.90, C_30_H_50_O_2_) with a pseudomolecular ion at *m*/*z* 443.3876 in positive ionization mode was putatively assigned as botulin. It was detected in both leaf and stem bark. It has shown fragment ions at *m*/*z* 425.37, 407.36, and 191.38 and they were similar to the report by Kosyakov et al. and Naumoska and Vovk [80,81]. 

#### 2.6.4. Identification of Fatty Acid Derivatives and Other Compounds

Five compounds (**63**, **70**, **71**, **72,** and **73**) in leaf and stem bark was detected and putatively assigned as fatty acid derivatives. Dicarboxylic fatty acids (compounds **63** and **71**) corresponded to pseudomoleculars [M-H]^-^ at *m*/*z* 187.0969 and *m*/*z* 215.1284, respectively showed fragment ions of [M-H-18]^−^, [M-H-28]^−^, and [M-H-46]^−^. Based on the fragmentation exhibited by these compounds, they were identified as azelaic acid (C_9_H_16_O_4_) and undecanedioic acid (C_11_H_20_O_4_) [82]. Trihydroxy-octadecadienoic acid (compounds **70**, **72** and **73**) were also detected at *m*/*z* 327.2179, *m*/*z* 327.2177, and *m*/*z* 329.2333 in the methanol extracts for leaf and stem bark. They revealed the same fragment ions at *m*/*z* 211.13 [M-H-C_6_H_12_O_2_]^−^ and 171.10 [M-H-C_9_H_14_-H_2_O]^−^. These fragment ions were also well-matched with those reported by Jiménez-Sánchez et al. [83].

#### 2.6.5. Identification of Other Compounds

Compound **1** (Rt = 0.66, C_7_H_12_O_6_) having a pseudomolecular ion at *m*/*z* 191.0553 in negative ionization mode was identified as quinic acid. It yielded fragment ions at *m*/*z* 171.03 [M-H-H_2_O]^−^, 127.04 [M-H-CO_2_-H_2_O]^−^ and 109.03 [M-H-CO_2_-H_2_O-H_2_O]^−^, similar to previous report [76]. Moreover, compound **2** (Rt = 0.93, C_12_H_16_O_7_), with a pseudomolecular ion at *m*/*z* 271.0801 in the negative mode, was identified as arbutin, which is a glycosylated hydroquinone. Its fragment ion at *m*/*z* 108.02 was due to a glucosyl moiety loss [84].

## 3. Materials and Methods 

### 3.1. Chemicals and Reagents

Ethanol, chloroform, methanol, ethyl acetate, hexane, formic acid, glacial acetic acid, 1-butanol, acetonitrile, sodium hydroxide (NaOH), hydrogen chloride (HCL), and dimethyl sulfoxide (DMSO) were purchased from Merck Millipore International (Darmstadt, Germany). The LCMS-grade acetonitrile, water, and formic acid were supplied by Fisher Scientific (Geel, Belgium). The sodium nitrate (NaNO_3_), iron(III) chloride (FeCl_3_), aluminum chloride hexahydrate (AlCl_3_·6H_2_O), ferrous sulfate heptahydrate (FeSO_4_·7H_2_O), gallic acid, sodium carbonate (Na_2_CO_3_), sodium acetate trihydrate, Folin-Ciocalteu reagent, 2, 2-diphenyl-1-picrylhydrazyl (DPPH), p-nitrophenyl-α-D-glucopyranose (PNPG), glycine, phosphate buffer, 2,4,6-tripyridyl-S-triazine (TPTZ) quercetin, and chlorohexidine were purchased from Sigma-Aldrich (St. Louis, MO, USA).

### 3.2. Plant Materials

The plant materials were obtained from Al Jabal, Al Akhdar (Green Mountain) region, North Eastern part of Libya and all samples were harvested during spring 2016. Plant authentication was carried out by Dr. Abdulamid Alzerbi of the Herbarium Unit, Department of Biology, Benghazi University, Libya. The leaf and stem barks of the plant were dried under shade for 15 and 28 days, respectively prior to mechanical grinding into a fine powder. The resulting particles were sieved using a stainless-steel sieve (80 mesh, Retsch, Haan, Germany) to obtain a fine and homogeneous powder. The finely powdered leaf and stem bark were weighed separately and all samples were stored at −20 °C (chiller) until needed. 

### 3.3. Preparation of Leaf and Stem Bark Extracts and Their Respective Fractions 

For extraction, the dried leaf (1500 g) and stem barks (500 g) were separately mixed with absolute methanol (CH_3_OH) with the solid to liquid ratio of 1:10 (*w*/*v*). The mixtures were subjected to sonication (at a controlled temperature) in an ultrasonic bath (Branson, 141 8510E-MTH models, Danbury, CT, USA) for an hour under a frequency of 53 KHz and power of 100 W. The extraction was repeated three times, each time with fresh solvent. The respective extracts were filtered using Whatman filter paper (GE Healthcare, Buckinghamshire, UK) and the collected filtrates were concentrated under reduced pressure using a rotary evaporator (Buchi, New Castle, DE, USA) at 40 °C. The leaf and stem bark methanolic extracts were then liquid–liquid partitioned, performed in a separating funnel, with solvents of increasing polarities, starting with hexane, chloroform, ethyl acetate and n-butanol, to yield the different fractions. In each case, the respective crude extract was resuspended in an adequate amount of methanol, and sonicated to aid the dissolution, before adding distilled water to bring the solution up to a workable volume for solvent fractionation. The extract solution was fractionated first with hexane at an extract solution:organic solvent ratio of 1:3. After vigorous shaking, the mixture was set aside until two layers were formed. The upper layer was collected as hexane fraction. To obtain the chloroform fraction, chloroform was added into the remaining fraction in the separating funnel, followed by vigorous shaking. The chloroform fraction was then collected. The same procedure was repeated using ethyl acetate and subsequently n-butanol to yield the ethyl acetate and n-butanol fractions. After partitioning with butanol, material left in the separating funnel was considered as the residual aqueous fraction. Workflow of the liquid–liquid partition of each of the crude extracts, together with information on yields and physical appearances of the extracts and their respective fraction, are shown in the Supplementary Information (Supplementary Figures S1 and S2, Supplementary Table S1). The solvent fractions were concentrated using rotary evaporator and finally lyophilized and kept in a chiller at −20 ℃ prior to further analysis. The aqueous fraction was not used for the analysis of the antioxidant and α-glucosidase inhibition due to the reason that it could not dissolve in the solvent used for sample preparation in the assays. 

### 3.4. Total Phenolic Content (TPC) Assay

The TPC in the methanolic extracts of *A. pavarii* leaf and stem bark as well as their respective fractions was determined using the Folin–Ciocalteu reagent, as described by Lee et al. [21], with slight modifications. An aliquot of 20 µL of extract or fraction at a concentration of (1000 μg/mL) was transferred into 96-well microplates, followed by 100 µL of the Folin-Ciocalteu reagent. After incubation at room temperature for 5 min, 80 µL of 7.5% sodium carbonate was added into the mixture and incubated for another 30 min. Finally, the absorbance was measured through a microplate reader (SPECTRAmax PLUS) at 765 nm. The analysis was performed in three replications for each sample. The standard curve of gallic acid was constructed to determine the TPC and the results were expressed as mg GAE/g extract for extracts and mg GAE/g fraction for fractions.

### 3.5. Total Flavonoid Content (TFC) Assay

The TFC was measured by using the colorimetric method as described by Kim et al. with slight modifications [23]. A volume of 25 µL of the extracts or fractions at a concentration of 1000 µL/mL were mixed with 100 µL distilled water and 7.5 µL 5% NaNO_2_ in a 96-well microplate. Then, 7.5 µL 10% AlCl_3_.6H_2_O was added and the resultant mixtures were incubated at room temperature. After 5 min, 50 µL 1 M NaOH was added into the mixture and the plate was incubated for another 15 min at room temperature. The absorbance was determined by using microplate reader at the wavelength of 415 nm. A standard curve of quercetin was used to calculate the TFC. The experiment was performed in triplicates and the TFC results were expressed as mg QE/g extract for extracts and mg QE/g fraction for fractions.

### 3.6. Free Radical Scavenging (DPPH) Assay

The DPPH assay was conducted in accordance with Lee et al. method [21]. Initially, 100 µL of DPPH (5.9 mg in 100 mL ethanol) was mixed with 50 µL of the extracts or fractions. The mixtures were then kept in dark for 30 min at room temperature. The absorbance was measured using microplate reader at 517 nm. Quercetin was used as a positive control in the assay. The scavenging activity was calculated using the equation Scavenging Activity (%) = [(Ac – As)/Ac] × 100%, where (Ac) is the absorbance of the blank reagent while (As) is the absorbance of the tested samples. The analysis was performed in triplicates and the results were described as IC_50_ value. 

### 3.7. Ferric Reducing Antioxidant Power (FRAP) Assay

The FRAP of extracts and fractions were examined according to Kadum et al. method with slight modifications [31]. The FRAP reagent was prepared by mixing the solutions of 2,4,6-tripyridyl-s-triazine (TPTZ) and FeCl_3_ in the acetic acid buffer (pH 3.6) in the ratio of 1:1:10 (*v*/*v*/*v*). A volume of 10 μL of methanol extract or fractions was transferred into 96-well microplates, followed by addition of 200 μL FRAP reagent and incubation at 37 °C for 30 min. The absorbance was checked through microplate reader (SPECTRAmax PLUS) at 593 nm. The absorbances of ferrous sulfate solution (FeSO_4_·7H_2_O), with a range of concentrations between 0.1 and 1 mM were used for plotting a calibration curve. The ascorbic acid was used as positive control and FRAP was presented as mM Fe (II)/g extract for extracts and mM Fe (II)/g fraction for fractions. 

### 3.8. α-Glucosidase Inhibitory Assay

The inhibitory activity of the extracts and fractions on α-glucosidase was assayed using method of Lee et al. with some slight modifications [21]. The α-glucosidase enzyme (0.02 U/well), and *p*-nitrophenyl-α-D-glucopyranose (PNPG) substrate (1 mM) were prepared in 50 mM phosphate buffer (pH 6.5). A total of 10 μL of extract or fraction was mixed with 130 μL of phosphate buffer (30 mM) and 10 μL of enzyme in a 96-well microplate. After incubation of the plate for 5 min, 50 μL of PNPG was added to each sample-containing well, blank substrate, negative control, and positive control, followed by further incubation for 15 min at room temperature to start the reaction. Subsequently, 50 μL of 2M glycine was added into each well to stop the reaction. The enzymatic activity was determined by calculating the p-nitrophenol that has been released from PNPG at the wavelength of 405 nm by using a spectrophotometer (SPECTRAmax PLUS, Sunnyvale, CA, USA). The inhibition percentage (%) was calculated using the following formula: % Inhibition = [(ΔAc – ΔAe)/ΔAc], where ΔAc is the absorbance difference between the negative control and blank control whereas ΔAe is the absorbance difference between sample and the blank sample. Eventually, the results were expressed as IC_50_ value in μg/mL.

### 3.9. UHPLC–MS/MS Analysis

The UHPLC–MS/MS analysis was conducted using a Dionex Ultimate 3000 UHPLC system attached to a Q Exactive^TM^ Focus Hybrid Quadrupole Orbitrap mass spectrometer (Thermo Scientific, San Jose, CA, USA). Analyte separation was carried out using a Hypersil Gold C18 column (2.1 × 100 mm, 1.9 µm, Thermo Scientific, San Jose, CA, USA) with a mobile phase consisting of LCMS grade water (solvent A) and acetonitrile (solvent B), each containing 0.1% formic acid and flowing at 0.4 mL/min. The programmed gradient consisted of 0 min (95% A), 5 min (95% A), 25 min (60% A), 55 min (0% A), 65 min (0% A), 67 min (95% A), and 70 min (95% A). Samples were prepared at the concentration of 1 mg/mL (*w*/*v*) by dissolving 1 mg of a dried sample of the active extract with the 1mL of LC–MS grade methanol. The resultant mixture was then filtered using 0.22 um nylon membranes and 10 µL of the filtrate was auto injected for the analysis. The MS analysis was done with the parameters set as follow: negative and positive ion mode (done separately), collision energy of 30 eV, spray voltage 4.2 kV (positive mode) and 3.5 kV (negative mode), capillary temperature 350 °C, auxiliary gas heater temperature 0 °C, sheath gas flow rate of 45 (arbitrary units) for the positive mode and 40 (arbitrary units) for the negative mode, and auxiliary nitrogen (99% pure) at a flow rate of 10 and 8 units for positive and negative mode, respectively. Then, the mass resolution was set to 70,000 full width at half maximum (FWHM) and a full scan from 150 to 2000 amu. The identification analysis was carried out by comparing between the available data of MS/MS from the literature.

### 3.10. Data Analysis

MS Excel (Version 2010), Minitab 16 software (Version 16, Minitab Inc., State College, PA, USA) were utilized for the analysis of the results of TPC, TFC, and biological activities. The results were expressed as the mean ± standard deviation of three replicates. One-way ANOVA with Tukey comparison test was employed to determine the significant difference among the samples. The *p* < 0.05 value was considered to be statistically significant, and vice versa. 

## 4. Conclusions

In this study, TPC and TFC, as well as the antioxidant and α-glucosidase inhibitory activities of methanol extracts and fractions of *A. pavarii* leaf and stem bark were examined. The phytochemical profiles of the methanol extracts of *A. pavarii* leaf and stem bark were also characterized using UHPLC–ESI–MS/MS. The results revealed that methanol extracts and fractions of *A. pavarii* exhibited different antioxidant and α-glucosidase inhibitory activities. Overall, the methanol extracts and polar fractions (ethyl acetate and butanol) exhibited remarkable TPC and TFC, as well as antioxidant and α-glucosidase inhibitory activities. In addition, plant phenolics, both flavonoids and non-flavonoid constituents, could be responsible for the antioxidant and α-glucosidase inhibitory activities of *A. pavarii* leaf and stem bark. Via the UHPLC–ESI–MS/MS analysis, a total of 76 compounds were putatively identified, in which a large proportion of them were phenolic compounds, which could be the constituents contributing to the antioxidant and α-glucosidase inhibitory activities of *A. pavarii* leaf and stem bark. However, the exact identity of the bioactive candidates for the two activities will require further rigorous investigations via bioassay-guided isolation and purification approach or other contemporary methods, such as multi-platform metabolomics. Nevertheless, the findings of this present study indicate the potential of *A. pavarii* as an antidiabetic agent. Further investigations of this therapeutical potential through in depth pharmacological studies, followed by research into its development as phytomedicinal preparations or health supplements for diabetes, will help pave the way towards its valorization. To the best of our knowledge, this study is the first report on the TPC, TFC, antioxidant activity, α-glucosidase inhibitory, and LC–MS/MS profiling for the stem bark of *A. pavarii*.

## Figures and Tables

**Table 1:** TPC, TFC, DPPH, FRAP, and α-glucosidase inhibition, activity of extracts and fractions of *A. pavarii*.

Extracts/Fraction/Standard	TPC (mg GAE/g Extract)	TFC (mg QE/g Extract)	DPPH IC_50_ (µg/mL)	FRAP (mmol Fe (II)/g Extract)	α-glycosidase IC_50_ (µg/mL)
**Leaf**					
**EX.MeOH**	886.57 ± 0.93 ^Aa^	442.06 ± 0.34 ^Ab^	17.57 ± 0.09 ^Aa^	82.95 ± 0.13 ^Aa^	8.75 ± 0.22 ^Ab^
**F.Hexane**	62.54 ± 0.27 ^Ba^	58.21 ± 0.06 ^Ba^	95.82 ± 0.20 ^Bb^	46.76 ± 0.57 ^Ba^	ND
**F.Chloroform**	186.94 ± 0.45 ^Ca^	109.09 ± 0.09 ^Cb^	39.50 ± 0.01 ^Ca^	50.43 ± 0.08 ^Ca^	62.64± 0.01 ^Ba^
**F.EtOAc**	790.76 ± 0.45 ^Da^	369.52 ± 0.47 ^Da^	6.39 ± 0. 30 ^Da^	86.33 ± 0.68 ^Da^	4.93 ± 0.09 ^Ca^
**F.BuOH**	390.47 ± 0.140 ^Eb^	277.72 ± 0.25 ^Ea^	27.69 ± 1.22 ^Eb^	82.17 ± 0.33 ^Aa^	10.44 ± 0.60 ^Db^
**Stem Bark**					
**EX.MeOH**	795.55 ± 1.45 ^Ab^	638.93 ± 0.29 ^Aa^	8.67 ± 0.45 ^Ab^	78.45 ± 0.61 ^Ab^	6.78 ± 0.08 ^Aa^
**F.Hexane**	199.14 ± 0.28 ^Bb^	38.41 ± 0.25 ^Bb^	77.07 ± 1.29 ^Ba^	37.59 ± 0.08 ^Bb^	ND
**F.Chloroform**	322.68 ± 0.01 ^Cb^	204.83 ± 0.34 ^Ca^	49.35 ± 0.01 ^Ca^	45.03 ± 0.75 ^Cb^	68.47 ± 0.06 ^Bb^
**F.EtOAc**	480.21± 0.13 ^Db^	255.463 ± 0.28 ^Db^	8.35 ± 0.29 ^Aa^	77.20 ± 0.32 ^Db^	5.05 ± 0.01 ^Ca^
**F.BuOH**	707.61± 0.58 ^Ea^	213.32 ±0.20 ^Eb^	8.71 ± 0.06 ^Aa^	79.19 ± 0.23 ^Ab^	5.90 ± 0.24 ^Ca^
**Quercetin**			8.6 ± 0.70		12.30 ± 1.40
**Ascorbic acid**				180.64 ± 0.01	

TPC, total phenolic content; TFC, total flavonoid content; DPPH, 2, 2-diphenyl-1-picrylhydrazyl; FRAP, ferric reducing antioxidant power; EX.MeOH, extract of methanol; F.Hexane, fraction of hexane; F.Chloroform, fraction of chloroform; F.EtOAc, fraction of ethyl acetate; F.BuOH, fraction of butanol. The experiment was conducted in triplicates and the result was calculated as mean ± SD. TPC assay, the curve absorbance of standard gallic acid versus sample concentrations is defined by equation y = 0.0036x + 0.0551(R^2^ = 0.9949). TFC assay, the curve absorbance of standard quercetin versus sample concentrations is defined by equation y = 0.0006x + 0.0326 (R^2^ = 0.9947). FRAP assay, the curve absorbance of standard ferrous sulfate versus sample concentrations is defined by equation y = 0.7708x + 0.0826 (R^2^ = 0.9987). The superscript uppercase letter is to compare the extracts and fractions of the same part. The superscript lowercase letter is to compare parts of the plant. ND = not determine.

**Table 2:** Compounds detected and identified in the leaf and stem bark methanol extracts of *A. pavarii*.

No	Retention Time, Rt (min)	Ionization Mode (+/-)	Experimental Parent Ion (*m*/*z*)	Theoretical Parent Ion (*m*/*z*)	Error (ppm)	MS/MS Fragment Ions	Compound Identity	Molecular Formula	Detection
**1**	0.66	-	191.0553	191.0555	−1.0468	171.03, 127.04, 109.03, 93.03	Quinic acid	C_7_H_12_O_6_	L, SB
**2**	0.93	-	271.0801	271.0817	−5.9022	252.90, 211.02, 108.02	Arbutin	C_12_H_16_O_7_	L, SB
**3**	1.00	-	331.0671	331.0665	1.8123	271.05, 211.02, 169.01	Gallic acid glucoside	C_13_H_16_O_10_	L, SB
**4**	1.05	-	331.0675	331.0665	3.0205	271.05, 211.02, 169.01	Gallic acid glucoside	C_13_H_16_O_10_	L, SB
**5**	1.24	-	331.0672	331.0665	2.1143	271.05, 211.02, 169.01	Gallic acid glucoside	C_13_H_16_O_10_	L, SB
**6**	1.25	-	169.0134	169.0137	−1.7750	125.02	Gallic acid	C_7_H_6_O_5_	L, SB
**7**	1.26	-	343.0668	343.0665	0.8744	191.06, 169.01	Galloylquinic acid	C_14_H_16_O_10_	L, SB
**8**	1.35	-	331.0669	331.0665	1.2082	271.05, 211.02, 169.01	Gallic acid glucoside	C_13_H_16_O_10_	L, SB
**9**	1.49	-	609.1246	609.1244	0.3283	483.10, 441.08, 423.07, 305.07, 177.02, 125.02	(Epi)gallocat+(epi)gallocat	C_30_H_26_O_14_	L, SB
**10**	1.96	-	315.0721	315.0716	1.5869	153.02, 152.01, 109.03, 108.02	Protocatechuic acid O-hexoside	C_13_H_16_O_9_	L, SB
**11**	1.98	-	329.0878	329.0872	1.8232	167.03, 152.01, 123.04, 108.02	Vanillic acid-*O*-glucoside ester	C_14_H_18_O_9_	L
**12**	2.04	-	329.0875	329.0872	0.9116	167.03, 152.01, 123.04, 108.02	Vanillic acid-*O*-glucoside ester	C_14_H_18_O_9_	L
**13**	2.10	-	305.06638	305.06613	0.81949	261.08, 179.03,167.03, 138.03, 137.02, 125.02	(Epi)gallocatechin	C_15_H_14_O_7_	L, SB
**14**	2.24	-	897.1866	897.1878	−1.3375	729.15, 711.14, 593.13, 407.08, 289.07	(Epi)gallocat+(epi)gallocat+(epi)cat	C_45_H_38_O_20_	SB
**15**	2.30	-	325.0566	325.0559	2.1534	169.01, 125.02	Galloyl shikimic acid	C_14_H_14_O_9_	SB
**16**	2.60	-	451.1254	451.1240	1.1083	289.07, 245.08, 151.04, 125.02	(Epi)catechin -3-*O*-glucoside	C_21_H_24_O_11_	L, SB
**17**	2.72	-	593.1291	593.1295	−0.6743	467.10, 441.08, 425.09, 407.08, 289.07, 177.02, 152.02	(Epi)gallocat+(epi)cat	C_30_H_26_O_13_	L, SB
**18**	3.04	-	359.0981	359.0978	0.8354	197.04, 153.05	Syringic acid-*O*-glucoside	C_15_H_20_O_10_	L, SB
**19**	3.62	-	881.1970	881.1929	4.6527	695.14, 577.14, 425.09, 407.08,303.05, 289.07, 125.02	(Epi)gallocat+(epi)cat+(epi)cat	C_45_H_38_O_19_	SB
**20**	5.04	-	451.1250	451.1240	2.2166	289.07, 245.08151.04, 125.02	(Epi)cat-3-*O*-glucoside	C_21_H_24_O_11_	SB
**21**	5.66	-	593.1314	593.1295	3.2033	467.10, 425.09, 407.08, 289.07, 177.02, 152.02	(Epi)gallocat+(epi)cat	C_30_H_26_O_13_	L, SB
**22**	5.94	-	577.1348	577.1346	0.3465	451.10, 425.09, 407.08, 289.07, 287.06, 245.08, 125.02	(Epi)cat+(epi)cat	C_30_H_26_O_12_	L, SB
**23**	6.81	-	451.1241	451.1240	0.2216	289.07, 245.08, 151.04, 125.02	(Epi)catechin -3-*O*-glucoside	C_21_H_24_O_11_	L, SB
**24**	7.01	-	289.0714	289.0712	0.67457	271.06, 245.08, 179.03, 165.02, 150.03, 137.02, 125.02	Catechin	C_15_H_14_O_6_	L, SB
**25**	7.51	-	865.1985	865.1979	0.6934	739.18, 713.15, 695.14, 577.13, 425.09, 407.08, 287.06, 125.02	(Epi)cat+(epi)cat +(epi)cat	C_45_H_38_O_18_	L, SB
**26**	7.56	-	483.0776	483.0774	0.4140	439.09, 424.54, 331.07, 313.06, 287.08, 271.05, 211.02, 169.01	Digalloyl glucoside	C_20_H_20_O_14_	L, SB
**27**	8.08	-	1153.2627	1153.2613	1.2139	865.20, 577.14, 407.08, 289.07, 125.02	(Epi)cat+(epi)cat +(epi)cat +(epi)cat	C_60_H_50_O_24_	SB
**28**	8.24	-	865.1973	865.1979	−0.6934	739.17, 713.15, 695.14, 577.13, 425.09, 407.08, 287.06, 125.02	(Epi)cat+(epi)cat +(epi)cat	C_45_H_38_O_18_	L, SB
**29**	9.00	-	319.0462	319.0453	2.8209	301.04, 193.01, 165.02, 153.02, 151.00, 137.02, 109.03	Dihydromyricetin	C_15_H_12_O_8_	L, SB
**30**	9.35	-	881.1908	881.1929	−2.3831	695.14, 577.14, 425.09, 407.08, 303.05, 289.07, 125.02	(Epi)gallocat+(epi)cat+(epi)cat	C_45_H_38_O_19_	SB
**31**	9.84	-	577.1356	577.1346	1.7326	451.10, 425.09, 407.08, 289.07, 287.06, 245.08, 125.02	(Epi)cat+(epi)cat	C_30_H_26_O_12_	L, SB
**32**	9.86	-	289.0717	289.0712	1.85075	271.06, 245.08, 179.03, 165.02, 150.03, 137.02, 125.02	Epicatechin	C_15_H_14_O_6_	L, SB
**33**	9.99	-	1017.2080	1017.2089	−0.8847	865.19, 577.14, 407.08 289.07, 125.02	(Epi)cat gallate +(epi)cat+(epi)cat	C_52_H_42_O_22_	SB
**34**	10.30	-	1017.2085	1017.2089	−0.3932	865.19, 577.14, 407.08 289.07, 125.02	(Epi)cat gallate +(epi)cat+(epi)cat	C_52_H_42_O_22_	L, SB
**35**	10.35	-	449.1087	449.1083	0.8906	287.06, 269.05, 243.07,151.00,107.01	Dihydrokaempferol 3- *O* –glucoside	C_21_H_22_O_11_	L, SB
**36**	10.42	-	635.0889	635.0884	0.7872	465.07, 313.06, 211.02, 169.01	Tigalloyl glucoside	C_27_H_24_O_18_	L, SB
**37**	10.45	-	1153.2617	1153.2613	0.3468	865.20, 577.14, 407.08, 289.07, 125.02	(Epi)cat+(epi)cat +(epi)cat +(epi)cat	C_60_H_50_O_24_	SB
**38**	10.53	-	729.1461	729.1455	0.8228	577.14, 559.13, 451.10, 425.09, 407.08, 289.07, 125.02	(Epi)catechgallate + (epi)cat	C_37_H_30_O_16_	L, SB
**39**	10.70	-	865.1970	865.1979	−1.0402	739.17, 713.15, 695.14, 577.14, 425.09, 407.08, 287.06, 125.02	(Epi)cat+(epi)cat +(epi)cat	C_45_H_38_O_18_	L, SB
**40**	11.06	-	479.0828	479.0825	0.6261	317.03, 316.02, 287.02, 271.02, 179.00, 151.00	Myricetin 3-*O*-glucoside	C_21_H_20_O_13_	SB
**41**	11.21	-	1153.2635	1153.2613	1.9076	865.20, 577.14, 407.08, 289.07, 125.02	(Epi)cat+(epi)cat+(epi)cat+(epi)cat	C_60_H_50_O_24_	SB
**42**	11.33	-	577.1369	577.1346	3.9852	451.10,425.09, 407.08, 289.07, 287.06, 245.05, 125.02	(Epi)cat+(epi)cat	C_30_H_26_O_12_	L, SB
**43**	11.42	-	729.1481	729.1455	3.5658	577.11, 407.08, 425.09, 289.07, 125.02	(Epi)catechgallate + (epi)cat	C_37_H_30_O_16_	L, SB
**44**	11.62	-	1017.2098	1017.2089	0.8847	865.19, 577.14, 407.08 289.07, 125.02	(Epi)catechgallate +(epi)cat+(epi)cat	C_52_H_42_O_22_	SB
**45**	12.15	-	615.0997	615.0986	1.7883	463.09, 300.03, 301.03, 271.02, 179.00, 151.00, 169.01	Quercetin-*O*-galloyl-glucoside	C_28_H_24_O_16_	L, SB
**46**	12.30	-	463.0878	463.0876	0.4318	317.03, 316.02, 287.02, 271.02, 179.00, 151.00	Myricetin 3-*O*-rhamnoside	C_21_H_20_O_12_	SB
**47**	12.49	-	300.9991	300.9984	2.3255	284.00, 257.00, 229.01, 201.02, 185.02	Ellagic acid	C_14_H_6_O_8_	L, SB
**48**	12.91	-	609.1456	609.1455	0.1641	301.03, 300.03, 271.02, 255.03	Rutin (quercetin-3-*O*-rutinoside)	C_27_H_30_O_16_	L, SB
**49**	12.96	+	465.1027	465.1033	−1.2900	303.05, 257.04, 229.05	Delphinidin-3-*O-* galacatoside	C_21_H_21_O_12_	L, SB
**50**	12.97	-	441.08258	441.0821	0.91819	289.07, 245.08, 203.07, 169.01	(Epi)catechin gallate	C_22_H_18_O_10_	L, SB
**51**	13.02	-	463.0885	463.0876	1.9434	301.03, 300.03, 271.07, 255.03	Quercetin-3-*O*-glucoside	C_21_H_20_O_12_	L, SB
**52**	13.18	+	465.0996	465.1033	−7.9552	303.05, 257.04, 229.05	Delphinidin-3-*O*-glucoside	C_21_H_21_O_12_	L, SB
**53**	13.21	+	449.1096	449.1083	2.8946	287.05, 241.00	Cyanidin-3-*O*-glucoside	C_21_H_21_O_11_	L, SB
**54**	13.43	-	593.1520	593.1506	2.3602	285.04, 284.03, 255.03, 227.03, 179.00, 151.00	kaempferol 7-*O*-rhamnosyl-(1→6)-glucoside	C_27_H_30_O_15_	SB
**55**	13.52	-	729.1456	729.1455	0.1371	577.14, 559.13, 451.11, 425.09, 407.08, 425.09, 289.07, 125.02	(Epi)catechgallate + (epi)cat	C_37_H_30_O_16_	L, SB
**56**	13.80	-	561.1404	561.1396	1.4256	451.11, 435.11, 289.07, 271.06	((Epi)afz + (epi)cat)	C_30_H_25_O_11_	L, SB
**57**	13.83	-	477.1042	477.1033	1.8863	315.05, 314.04, 285.04, 271.02, 243.03	Isorhamnetin 3-*O*-glucoside	C_22_H_22_O_12_	SB
**58**	13.94	-	433.0773	433.0770	0.6927	301.03, 300.03, 271.02, 255.03	Quercetin-3-*O*-(arabinoside/xyloside)	C_20_H_18_O_11_	L, SB
**59**	13.98	-	447.0936	447.0927	2.0130	285.04, 284.03, 255.03, 227.03	kaempferol-3-*O*-glucoside	C_21_H_20_O_11_	L, SB
**60**	14.43	-	317.0310	317.0297	4.1005	289.07, 179.00, 151.00	Myricetin	C_15_H_10_O_8_	SB
**61**	14.45	+	479.1156	479.1189	−6.8876	317.06, 302.03, 274.04, 257.04	Petunidin-3-*O*-glucoside	C_22_H_23_O_12_	L, SB
**62**	14.53	-	447.0935	447.0927	1.7893	301.03, 300.03, 271.02, 255.03	Quercetin-3-*O*-rhamnoside	C_21_H_20_O_11_	L, SB
**63**	14.73	-	187.0969	187.0970	−0.5344	169.09, 143.11, 125.10	Azelaic acid	C_9_H_16_O_4_	L, SB
**64**	15.61	-	583.1099	583.1087	2.0579	463.09, 301.03, 300.03, 271.03, 255.03	Quercetin-*O*-(p-hydroxy)benzoyl-hexoside	C_28_H_24_O_14_	L, SB
**65**	16.04	+	609.1611	609.1608	0.4924	301.07, 286.04, 258.05	Peonidin-3-*O*-(p-coumaroyl- glucoside)	C_31_H_29_O_13_	L, SB
**66**	16.21	-	435.1295	435.1291	0.9192	273.08, 167.03, 123.04	Phloridzin	C_21_H_24_O_10_	SB
**67**	16.23	+	463.1203	463.1240	−7.9892	301.07, 286.04, 258.05	Peonidin-3-*O*-glucoside	C_22_H_23_O_11_	L, SB
**68**	16.72	+	493.1328	493.1346	−3.6501	331.08, 316.05, 315.04, 287.12	Malvidin-3-*O*-glucoside	C_23_H_25_O_12_	L, SB
**69**	17.77	-	301.0353	301.0348	1.6609	271.02, 255.03, 179.00, 151.00, 149.02, 121.03, 121.03,107.01	Quercetin	C_15_H_10_O_7_	L, SB
**70**	19.79	-	327.2179	327.2171	2.4448	229.14, 211.13, 171.10	Trihydroxy-octadecadienoic acid	C_18_H_32_O_5_	SB
**71**	20.39	-	215.1284	215.1283	0.4648	197.12, 153.13	Undecanedioic acid	C_11_H_20_O_4_	L, SB
**72**	21.03	-	327.2177	327.2171	1.8336	229.14, 211.13, 171.10	Trihydroxy-octadecadienoic acid	C_18_H_32_O_5_	L, SB
**73**	22.43	-	329.2333	329.2328	1.51868	229.14, 211.13, 171.10	Trihydroxy-octadecadienoic acid	C_18_H_34_O_5_	L, SB
**74**	40.99	+	457.3679	457.3681	−0.4372	439.35, 411.36, 393.35, 203.18, 189.16	Betulinic acid	C_30_H_48_O_3_	L, SB
**75**	41.17	+	457.3653	457.3681	−6.1219	439.35, 411.36, 393.35, 249.18, 203.18, 133.10	Ursolic acid	C_30_H_48_O_3_	L, SB
**76**	42.90	+	443.3876	443.3889	−2.9319	425.37, 407.36, 191.38	Betulin	C_30_H_50_O_2_	L, SB

Cat, catechin; Ep, epicatechin; afz, afzelechin; L, leaf; SB, stem bark.

## Data Availability

Data is contained within the article or Supplementary Material.

## Supplementary Materials

The following are available online at https://www.mdpi.com/article/10.3390/plants10081659/s1, Figure S1: Fractionation of crude methanolic extract of *A. pavarii* leaf, Figure S2: Fractionation of crude methanolic extract of *A. pavarii* stem bark, Table S1: Yield of extracts and fractions of *Arbutus pavarii*.

## Sample Availability

Samples of the compounds are available from the authors.

## References

[1] Chaturvedi D., Singh K., Singh V.K. (2019). Therapeutic and pharmacological aspects of photodynamic product chlorophyllin. Eur. J. Biol. Res..

[2] Joshi B.K., Shrestha R., Gautam I.P., Poudel A.P., Gotame T.P. (2019). Neglected and Underutilized Species (NUS), and Future Smart Food (FSF) in Nepal.

[3] Amin M.U., Khurram M., Khattak B., Khan J. (2015). Antibiotic additive and synergistic action of rutin, morin and quercetin against methicillin resistant *Staphylococcus aureus*. BMC Complement Altern. Med..

[4] Riani M.K.L., Anwar E., Nurhayati T. (2018). Antioxidant and anti-collagenase activity of sargassum plagyophyllum extract as an anti-wrinkle cosmetic ingredient. Pharmacogn. J..

[5] Zhang Y.J., Gan R.Y., Li S., Zhou Y., Li A.N., Xu D.P., Li H.B. (2015). Antioxidant phytochemicals for the prevention and treatment of chronic diseases. Molecules.

[6] Lourenço S.C., Moldão-Martins M., Alves V.D. (2019). Antioxidants of natural plant origins: From sources to food industry applications. Molecules.

[7] Pinaffi A.C.D.C., Sampaio G.R., Soares M.J., Shahidi F., de Camargo A.C., Torres E.A. (2020). Insoluble-Bound Polyphenols Released from Guarana Powder: Inhibition of Alpha-Glucosidase and Proanthocyanidin Profile. Molecules.

[8] Lobo V., Patil A., Phatak A., Chandra N. (2010). Free radicals, antioxidants and functional foods: Impact on human health. Pharmacogn. Rev..

[9] Tan Y., Chang S.K., Zhang Y. (2017). Comparison of α-amylase, α-glucosidase and lipase inhibitory activity of the phenolic substances in two black legumes of different genera. Food Chem..

[10] Malik A., Jamil U., Butt T.T., Waquar S., Gan S.H., Shafique H., Jafar T.H. (2019). In silico and in vitro studies of lupeol and iso-orientin as potential antidiabetic agents in a rat model. Drug Des. Dev. Ther..

[11] Alkhalidy H., Wang Y., Liu D. (2018). Dietary flavonoids in the prevention of T2D: An overview. Nutrients.

[12] Tundis R., Loizzo M.R., Menichini F. (2010). Natural products as α-amylase and α-glucosidase inhibitors and their hypoglycaemic potential in the treatment of diabetes: An update. Mini-Rev. Med..

[13] Hegazy A., Boulos L., Kabiel H., Sharashy O. (2011). Vegetation and species altitudinal distribution in Al-Jabal Al-Akhdar landscape, Libya. Pak. J. Bot..

[14] Alghazeer R., Abourghiba T., Ibrahim A., Zreba E. (2016). Bioactive properties of some selected Libyan plants. J. Med. Plant Res..

[15] Tenuta M.C., Tundis R., Xiao J., Loizzo M.R., Dugay A., Deguin B. (2019). *Arbutus* species (*Ericaceae*) as source of valuable bioactive products. Crit. Rev. Food Sci..

[16] Buzgaia N., Awin T., Elabbar F., Abdusalam K., Lee S.Y., Rukayadi Y., Abas F., Shaari K. (2020). Antibacterial Activity of Arbutus pavarii Pamp against Methicillin-Resistant Staphylococcus aureus (MRSA) and UHPLC-MS/MS Profile of the Bioactive Fraction. Plants.

[17] El Shibani F.A.E.S. (2017). A Pharmacognostical Study of *Arbutus pavarii* Pampan. Family Ericaceae and *Sarcopoterium spinosum* L. Family Rosaceae Growing in Libya. Ph.D. Thesis.

[18] de Camargo A.C., Favero B.T., Morzelle M.C., Franchin M., Alvarez-Parrilla E., de la Rosa L.A., Geraldi M.V., Marostica Junior M.R., Shahidi F., Schwember A.R. (2019). Is chickpea a potential substitute for soybean? Phenolic bioactives and potential health benefits. Int. J. Mol. Sci..

[19] de Camargo A.C., Biasoto A.C.T., Schwember A.R., Granato D., Rasera G.B., Franchin M., Rosalen P.L., Alencar S.M., Shahidi F. (2019). Should we ban total phenolics and antioxidant screening methods? The link between antioxidant potential and activation of NF-κB using phenolic compounds from grape by-products. Food Chem..

[20] Falcão H.G., Silva M.B.R., de Camargo A.C., Shahidi F., Franchin M., Rosalen P.L., Alencar S.M., Kurozawa L.E., Ida E.I. (2019). Optimizing the potential bioactivity of isoflavones from soybeans via ultrasound pretreatment: Antioxidant potential and NF--κB activation. J. Food Biochem..

[21] Lee S.Y., Mediani A., Nur Ashikin A.H., Azliana A.B.S., Abas F. (2014). Antioxidant and α-glucosidase inhibitory activities of the leaf and stem of selected traditional medicinal plants. Int. Food Res. J..

[22] Truong D.H., Nguyen D.H., Ta N.T.A., Bui A.V., Do T.H., Nguyen H.C. (2019). Evaluation of the use of different solvents for phytochemical constituents, antioxidants, and in vitro anti-inflammatory activities of *Severinia buxifolia*. J. Food Qual..

[23] Kim D.O., Jeong S.W., Lee C.Y. (2003). Antioxidant capacity of phenolic phytochemicals from various cultivars of plums. Food Chem..

[24] Deng Y., Zhao Y., Padilla-Zakour O., Yang G. (2019). Polyphenols, antioxidant and antimicrobial activities of leaf and bark extracts of *Solidago canadensis* L.. Ind. Crops Prod..

[25] Balasundram N., Sundram K., Samman S. (2006). Phenolic compounds in plants and agri-industrial by-products: Antioxidant activity, occurrence, and potential uses. Food Chem..

[26] Alam M.A., Zaidul I.S.M., Ghafoor K., Sahena F., Hakim M.A., Rafii M.Y., Abir H.M., Bostanudin M.F., Perumal V., Khatib A. (2017). In vitro antioxidant and, α-glucosidase inhibitory activities and comprehensive metabolite profiling of methanol extract and its fractions from *Clinacanthus nutans*. BMC Compl. Altern. Med..

[27] Apak R., Güclü K., Özyürek M., Celik S.E. (2008). Mechanism of antioxidant capacity assays and the CUPRAC (cupric ion reducing antioxidant capacity) assay. Microchim. Acta.

[28] Aruoma O.I. (2003). Methodological considerations for characterizing potential antioxidant actions of bioactive components in plant foods. Mutat. Res..

[29] Noreen H., Semmar N., Farman M., McCullagh J.S. (2017). Measurement of total phenolic content and antioxidant activity of aerial parts of medicinal plant *Coronopus didymus*. Asian Pac. J. Trop. Med..

[30] Nakamura M., Ra J.H., Jee Y., Kim J.S. (2017). Impact of different partitioned solvents on chemical composition and bioavailability of Sasa quelpaertensis Nakai leaf extract. J. Food Drug Anal..

[31] Kadum H., Hamid A.A., Abas F., Ramli N.S., Mohammed A.K.S., Muhialdin B.J., Jaafar A.H. (2019). Bioactive Compounds Responsible for Antioxidant Activity of Different Varieties of Date (*Phoenix dactylifera* L.) Elucidated by ^1^H-NMR Based Metabolomics. Int. J. Food Prop..

[32] Brown A., Anderson D., Racicot K., Pilkenton S.J., Apostolidis E. (2017). Evaluation of phenolic phytochemical enriched commercial plant extracts on the in vitro inhibition of α-glucosidase. Front. Nutr..

[33] Ganzera M., Sturm S. (2018). Recent advances on HPLC/MS in medicinal plant analysis—An update covering 2011–2016. J. Pharm. Biomed. Anal..

[34] Mendes L., de Freitas V., Baptista P., Carvalho M. (2011). Comparative antihemolytic and radical scavenging activities of strawberry tree (*Arbutus unedo* L.) leaf and fruit. Food Chem. Toxicol..

[35] Abu-Reidah I.M., Ali-Shtayeh M.S., Jamous R.M., Arráez-Román D., Segura-Carretero A. (2015). HPLC–DAD–ESI–MS/MS screening of bioactive components from *Rhus coriaria* L. (Sumac) fruits. Food Chem..

[36] Liu Y., Seeram N.P. (2018). Liquid chromatography coupled with time-of-flight tandem mass spectrometry for comprehensive phenolic characterization of pomegranate fruit and flower extracts used as ingredients in botanical dietary supplements. J. Sep. Sci..

[37] Karar M., Kuhnert N. (2015). UPLC-ESI-Q-TOF-MS/MS characterization of phenolics from *Crataegus monogyna* and *Crataegus laevigata* (Hawthorn) leaf, fruits and their herbal derived drops (Crataegutt Tropfen). J. Chem. Biol. Therap..

[38] Morales-Soto A., Gómez-Caravaca A.M., García-Salas P., Segura-Carretero A., Fernández-Gutiérrez A. (2013). High-performance liquid chromatography coupled to diode array and electrospray time-of-flight mass spectrometry detectors for a comprehensive characterization of phenolic and other polar compounds in three pepper (*Capsicum annuum* L.) samples. Food Res. Int..

[39] Gutiérrez-Grijalva E.P., Picos-Salas M.A., Leyva-López N., Criollo-Mendoza M.S., Vazquez-Olivo G., Heredia J.B. (2018). Flavonoids and phenolic acids from oregano: Occurrence, biological activity and health benefits. Plants.

[40] Nijveldt R.J., Van Nood E.L.S., Van Hoorn D.E., Boelens P.G., Van Norren K., Van Leeuwen P.A. (2001). Flavonoids: A review of probable mechanisms of action and potential applications. Am. J. Clin. Nutr..

[41] Thilakarathna S.H., Wang Y., Rupasinghe H.V., Ghanam K. (2012). Apple peel flavonoid-and triterpene-enriched extracts differentially affect cholesterol homeostasis in hamsters. J. Funct. Foods..

[42] Tungmunnithum D., Thongboonyou A., Pholboon A., Yangsabai A. (2018). Flavonoids and other phenolic compounds from medicinal plants for pharmaceutical and medical aspects: An overview. Medicines.

[43] Singh A., Kumar S., Kumar B. (2018). LC-MS Identification of Proanthocyanidins in Bark and Fruit of six Terminalia species. Nat. Prod. Commun..

[44] Stöggl W.M., Huck C.W., Bonn G.K. (2004). Structural elucidation of catechin and epicatechin in sorrel leaf extracts using liquid--chromatography coupled to diode array--, fluorescence--, and mass spectrometric detection. J. Sep. Sci..

[45] Yuzuak S., Ballington J., Xie D.Y. (2018). HPLC-qTOF-MS/MS-Based Profiling of Flavan-3-ols and Dimeric Proanthocyanidins in Berries of Two Muscadine Grape Hybrids FLH 13-11 and FLH 17-66. Metabolites.

[46] Jeszka-Skowron M., Zgoła-Grześkowiak A., Frankowski R. (2018). Cistus incanus a promising herbal tea rich in bioactive compounds: LC–MS/MS determination of catechins, flavonols, phenolic acids and alkaloids—A comparison with Camellia sinensis, Rooibos and Hoan Ngoc herbal tea. J. Food Compost. Anal..

[47] Di Lecce G., Arranz S., Jáuregui O., Tresserra-Rimbau A., Quifer-Rada P., Lamuela-Raventós R.M. (2014). Phenolic profiling of the skin, pulp and seeds of Albariño grapes using hybrid quadrupole time-of-flight and triple-quadrupole mass spectrometry. Food Chem..

[48] Munekata P.E.S., Franco D., Trindade M.A., Lorenzo J.M. (2016). Characterization of phenolic composition in chestnut leaf and beer residue by LC–DAD–ESI–MS. LWT-Food Sci. Technol..

[49] Ismail B.B., Pu Y., Guo M., Ma X., Liu D. (2019). LC-MS/QTOF identification of phytochemicals and the effects of solvents on phenolic constituents and antioxidant activity of baobab (*Adansonia digitata*) fruit pulp. Food Chem..

[50] Wang C., Li Q., Han G., Zou L., Lv L., Zhou Q., Li N. (2010). LC MS/MS for simultaneous determination of four major active catechins of tea polyphenols in rat plasma and its application to pharmacokinetics. Chin. Herb. Med..

[51] Gođevac D., Jadranin M., Aljančić I., Vajs V., Tešević V., Milosavljević S. (2015). Application of Spectroscopic Methods and Hyphenated Techniques to the Analysis of Complex Plant Extracts. Medicinal and Aromatic Plants of the World.

[52] Bystrom L.M., Lewis B.A., Brown D.L., Rodriguez E., Obendorf R.L. (2008). Characterisation of phenolics by LC–UV/Vis, LC–MS/MS and sugars by GC in *Melicoccus bijugatus* Jacq. ‘Montgomery’fruits. Food Chem..

[53] Friedrich W., Eberhardt A., Galensa R. (2000). Investigation of proanthocyanidins by HPLC with electrospray ionization mass spectrometry. Eur. Food Res. Technol..

[54] Gu L., Kelm M.A., Hammerstone J.F., Beecher G., Holden J., Haytowitz D., Prior R.L. (2003). Screening of foods containing proanthocyanidins and their structural characterization using LC-MS/MS and thiolytic degradation. J. Agric. Food Chem..

[55] Delcambre A., André Y., Saucier C. (2015). Sequencing of red wine proanthocyanidins by UHPLC-ESI-Q-TOF. J. Appl. Bioanal..

[56] Verardo V., Bonoli M., Marconi E., Caboni M.F. (2008). Determination of free flavan-3-ol content in barley (*Hordeum vulgare* L.) air-classified flours: Comparative study of HPLC-DAD/MS and spectrophotometric determinations. J. Agric. Food Chem..

[57] Jaiswal R., Jayasinghe L., Kuhnert N. (2012). Identification and characterization of proanthocyanidins of 16 members of the Rhododendron genus (*Ericaceae*) by tandem LC–MS. J. Mass Spectrom..

[58] Prasain J.K., Peng N., Dai Y., Moore R., Arabshahi A., Wilson L., Kim H., Watts R.L. (2009). Liquid chromatography tandem mass spectrometry identification of proanthocyanidins in rat plasma after oral administration of grape seed extract. Phytomedicine.

[59] Pallauf K., Rivas-Gonzalo J.C., Del Castillo M.D., Cano M.P., de Pascual-Teresa S. (2008). Characterization of the antioxidant composition of strawberry tree (*Arbutus unedo* L.) fruits. J. Food Compos. Anal..

[60] Rue E.A., Rush M.D., Van Breemen R.B. (2018). Procyanidins: A comprehensive review encompassing structure elucidation via mass spectrometry. Phytochem. Rev..

[61] Rockenbach I.I., Jungfer E., Ritter C., Santiago-Schübel B., Thiele B., Fett R., Galensa R. (2012). Characterization of flavan-3-ols in seeds of grape pomace by CE, HPLC-DAD-MSn and LC-ESI-FTICR-MS. Food Res. Int..

[62] Hamed A.I., Al--Ayed A.S., Moldoch J., Piacente S., Oleszek W., Stochmal A. (2014). Profiles analysis of proanthocyanidins in the argun nut (*Medemia argun*—An ancient Egyptian palm) by LC–ESI–MS/MS. J. Mass Spectrom..

[63] Nawrot-Hadzik I., Ślusarczyk S., Granica S., Hadzik J., Matkowski A. (2019). Phytochemical Diversity in Rhizomes of Three Reynoutria Species and their Antioxidant Activity Correlations Elucidated by LC–ESI–MS/MS Analysis. Molecules.

[64] Downey M.O., Rochfort S. (2008). Simultaneous separation by reversed-phase high-performance liquid chromatography and mass spectral identification of anthocyanins and flavonols in Shiraz grape skin. J. Chromatogr. A.

[65] Trikas E., Papi R., Kyriakidis D., Zachariadis G. (2016). A sensitive LC-MS method for anthocyanins and comparison of byproducts and equivalent wine content. Separations.

[66] Kajdžanoska M., Gjamovski V., Stefova M. (2010). HPLC–DAD–ESI–MSn identification of phenolic compounds in cultivated strawberries from Macedonia. Maced. J. Chem. Chem. Eng..

[67] Veberic R., Slatnar A., Bizjak J., Stampar F., Mikulic-Petkovsek M. (2015). Anthocyanin composition of different wild and cultivated berry species. LWT-Food Sci. Technol..

[68] Stein-Chisholm R., Beaulieu J., Grimm C., Lloyd S. (2017). LC–MS/MS and UPLC–UV evaluation of anthocyanins and anthocyanidins during rabbiteye blueberry juice processing. Beverages.

[69] Sun J., Liang F., Bin Y., Li P., Duan C. (2007). Screening non-colored phenolics in red wines using liquid chromatography/ultraviolet and mass spectrometry/mass spectrometry libraries. Molecules.

[70] Hvattum E., Ekeberg D. (2003). Study of the collision-induced radical cleavage of flavonoid glycosides using negative electrospray ionization tandem quadrupole mass spectrometry. J. Mass Spectrom..

[71] Singh A.P., Wang Y., Olson R.M., Luthria D., Banuelos G.S., Pasakdee S., Vorsa N., Wilson T. (2017). LC-MS-MS analysis and the antioxidant activity of flavonoids from eggplant skins grown in organic and conventional environments. J. Food Sci. Nutr..

[72] Fan L., Tong Q., Dong W., Yang G., Hou X., Xiong W., Wang W. (2017). Tissue distribution, excretion, and metabolic profile of dihydromyricetin, a flavonoid from vine tea (*Ampelopsis grossedentata*) after oral administration in rats. J. Agric. Food Chem..

[73] Engström M.T., Pälijärvi M., Salminen J.P. (2015). Rapid fingerprint analysis of plant extracts for ellagitannins, gallic acid, and quinic acid derivatives and quercetin-, kaempferol-and myricetin-based flavonol glycosides by UPLC-QqQ-MS/MS. J. Agric. Food Chem..

[74] Zhu M.Z., Wu W., Jiao L.L., Yang P.F., Guo M.Q. (2015). Analysis of flavonoids in lotus (*Nelumbo nucifera*) leaf and their antioxidant activity using macroporous resin chromatography coupled with LC-MS/MS and antioxidant biochemical assays. Molecules.

[75] Sánchez-Rabaneda F., Jauregui O., Lamuela-Raventós R.M., Viladomat F., Bastida J., Codina C. (2004). Qualitative analysis of phenolic compounds in apple pomace using liquid chromatography coupled to mass spectrometry in tandem mode. Rapid Commun. Mass Spectrom..

[76] Kumar S., Chandra P., Bajpai V., Singh A., Srivastava M., Mishra D.K., Kumar B. (2015). Rapid qualitative and quantitative analysis of bioactive compounds from *Phyllanthus amarus* using LC/MS/MS techniques. Ind. Crops Prod..

[77] Hossain M.B., Rai D.K., Brunton N.P., Martin-Diana A.B., Barry-Ryan C. (2010). Characterization of phenolic composition in Lamiaceae spices by LC–ESI–MS/MS. J. Agric. Food Chem..

[78] Budzikiewicz H., Wilson J., Djerassi C. (1963). Mass spectrometry in structural and stereochemical problems. XXXII. 1 Pentacyclic triterpenes. J. Am. Chem. Soc..

[79] Huang L., Chen T., Ye Z., Chen G. (2007). Use of liquid chromatography–atmospheric pressure chemical ionization–ion trap mass spectrometry for identification of oleanolic acid and ursolic acid in *Anoectochilus roxburghii* (wall.) Lindl. Int. J. Mass Spectrom..

[80] Kosyakov D.S., Ul’Yanovskii N.V., Falev D.I. (2014). Determination of triterpenoids from birch bark by liquid chromatography-tandem mass spectrometry. J. Anal. Chem..

[81] Naumoska K., Vovk I. (2015). Analysis of triterpenoids and phytosterols in vegetables by thin-layer chromatography coupled to tandem mass spectrometry. J. Chromatogr. A..

[82] Jiménez-Sánchez C., Lozano-Sánchez J., Gabaldón-Hernández J.A., Segura-Carretero A., Fernández-Gutiérrez A. (2015). RP-HPLC–ESI–QTOF/MS2 based strategy for the comprehensive metabolite profiling of *Sclerocarya Birrea* (Marula) bark. Ind. Crops Prod..

[83] Jiménez-Sánchez C., Lozano-Sánchez J., Rodríguez-Pérez C., Segura-Carretero A., Fernández-Gutiérrez A. (2016). Comprehensive, untargeted, and qualitative RP-HPLC-ESI-QTOF/MS2 metabolite profiling of green asparagus (*Asparagus officinalis*). J. Food Compos. Anal..

[84] de la Luz Cádiz-Gurrea M., Fernández-Arroyo S., Joven J., Segura-Carretero A. (2013). Comprehensive characterization by UHPLC-ESI-Q-TOF-MS from an Eryngium bourgatii extract and their antioxidant and anti-inflammatory activities. Food Res. Int..

