# Supplementary material for: Antioxidant Activity, α-Glucosidase Inhibition and UHPLC–ESI–MS/MS Profile of Shmar (Arbutus pavarii Pamp)
Source: Plants (Basel). 2021 Aug 11;10(8):1659. doi: 10.3390/plants10081659 (PMC8398081; doi:10.3390/plants10081659)
Supplement: Supplementary file 1 [file plants-10-01659-s001.zip › plants-1181623-supplementary.pdf]

Supplementary Materials:

Antioxidant Activity, *α*-Glucosidase Inhibition and UHPLC–ESI–MS/MS Profile of Shmar (*Arbutus pavarii* Pamp)

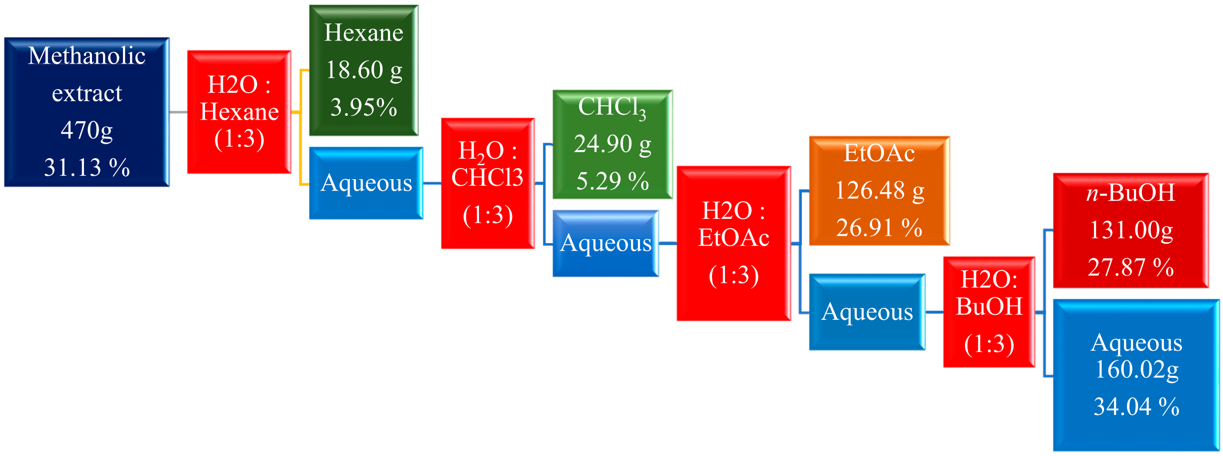

Figure S1. Fractionation of crude methanolic extract of *A.pavarii* leaf

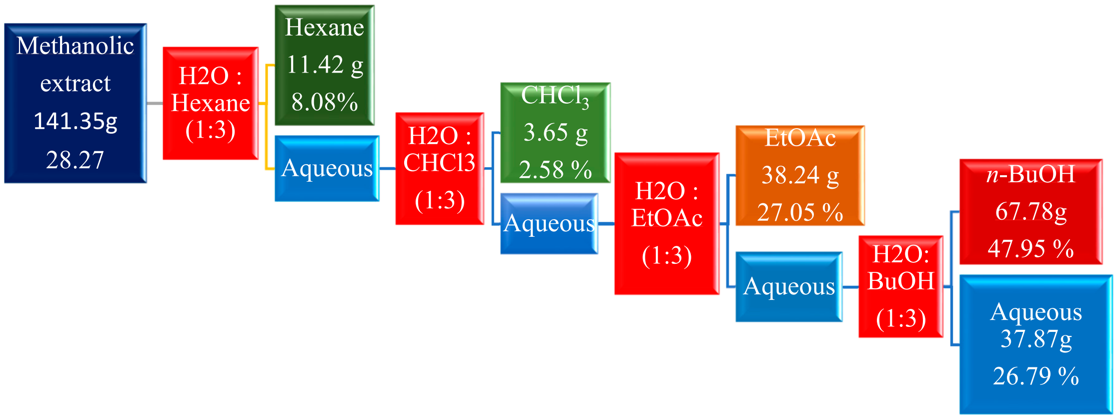

Figure S2. Fractionation of crude methanolic extract of *A.pavarii* stem bark.

Table S1. Yield of extracts and solvent fractions of *Arbutus pavarii*

Plant part	Solvent	Weight (g)	Yield %	Physical appearance
Leaf	CH_3OH	470	31.13	Dark-green brown gum
	Hex	18.60	3.95	Dark-green gum
	$CHCl_3$	24.90	5.29	Green gum
	EtOAc	126.48	26.91	Dark-orang gum
	n-BuOH	131.00	27.87	Brown gum
Stem bark	CH_3OH	141.35	28.27	green-brown gum
	Hex	11.42	8.08	Dark-green gum
	$CHCl_3$	3.65	2.58	Green gum
	EtOAc	38.24	27.05	Dark-brown gum
	n-BuOH	67.78	47.95	Dark-brown gum

CH_3OH, methanol; Hex, hexane; $CHCl_3$, chloroform; EtOAc, ethyl acetate; n-BuOH, butanol
